# Synthesis and Antagonist Activity of Methyllycaconitine Analogues on Human α7 Nicotinic Acetylcholine Receptors

**DOI:** 10.1021/acsbiomedchemau.2c00057

**Published:** 2023-02-14

**Authors:** Ashraf M. A. Qasem, Michael G. Rowan, Victoria R. Sanders, Neil S. Millar, Ian S. Blagbrough

**Affiliations:** ‡ School of Pharmacy, University of Bath, Bath BA2 7AY, U.K.; † Department of Neuroscience, Physiology and Pharmacology, University College London, Gower Street, London WC1E 6BT, U.K.

**Keywords:** antagonist, human α7 nAChR, methyllycaconitine (MLA), 2-methylsuccinimido benzoate ester, nicotinic acetylcholine receptors (nAChR), nicotinic competitive antagonist, norditerpenoid alkaloid

## Abstract

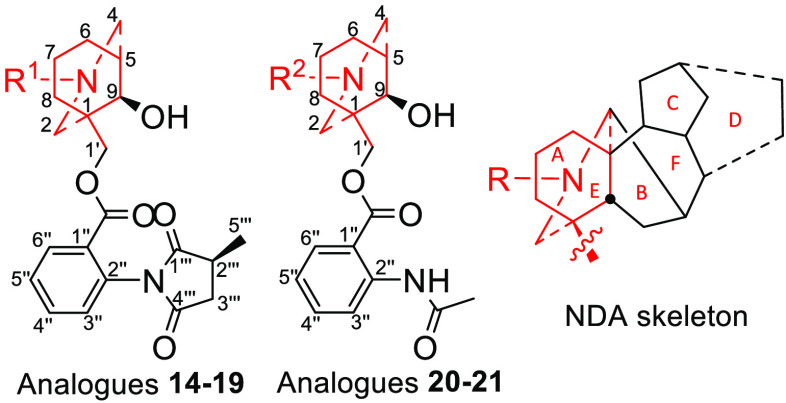

Methyllycaconitine (MLA), **1**, is a naturally occurring norditerpenoid alkaloid that is a highly potent (IC_50_ = 2 nM) selective antagonist of α7 nicotinic acetylcholine receptors (nAChRs). Several structural factors affect its activity such as the neopentyl ester side-chain and the piperidine ring N-side-chain. The synthesis of simplified AE-bicyclic analogues **14**–**21** possessing different ester and nitrogen side-chains was achieved in three steps. The antagonist effects of synthetic analogues were examined on human α7 nAChRs and compared to that of MLA **1**. The most efficacious analogue (**16**) reduced α7 nAChR agonist responses [1 nM acetylcholine (ACh)] to 53.2 ± 1.9% compared to 3.4 ± 0.2% for MLA **1**. This demonstrates that simpler analogues of MLA **1** possess antagonist effects on human α7 nAChRs but also indicates that further optimization may be possible to achieve antagonist activity comparable to that of MLA **1**.

## Introduction

Nicotinic acetylcholine receptors (nAChRs) are members of a superfamily of ligand-gated ion channels and are receptors for the neurotransmitter acetylcholine (ACh). They are oligomeric proteins in which five transmembrane subunits coassemble to form a central cation-selective pore. Agonists, such as ACh, bind to a site on the extracellular region of nAChRs and, in doing so, cause a conformational change in the receptor that results in the opening of the transmembrane ion channel and the influx of cations.^1,2^ Nicotinic receptors are located at postsynaptic sites (for example, on nerve and muscle cells), where they can mediate rapid neuronal or neuromuscular signaling, but they are also located at presynaptic sites (for example, in the brain), where they can play a more modulatory role. Sixteen nAChR subunits are expressed in humans (α1−α7, α9, α10, β1−β4, γ, δ, and ε) and these can coassemble into a diverse array of both homomeric and heteromeric nAChR subtypes with distinct physiological and pharmacological properties.^3^ One nAChR subtype that has attracted particular attention is the α7 nAChR, a homomeric receptor containing five copies of the α7 subunit. It is expressed in several regions of the brain and has been implicated in a range of neurological disorders.^4^

Signaling through nAChRs can be blocked by the binding of antagonists acting either at the orthosteric agonist binding site (competitive antagonists) or at distinct allosteric sites (noncompetitive antagonists).^5^ Methyllycaconitine (MLA), **1**, is one example of a nAChR competitive antagonist that is highly potent and highly selective for α7 nAChRs.^6^ It forms part of a broader family of norditerpenoid alkaloids (NDAs) from *Delphinium* and *Aconitum*, which are highly oxygenated hexacyclic systems and can exert a variety of pharmacological effects by modulating transmembrane proteins such as nAChRs and voltage gated sodium channels (VGSCs).^7,8^ In addition, the potential therapeutic use of MLA **1** has been examined in connection with disorders such as cerebral palsy and Parkinson’s disease.^8^ MLA **1** and other *Delphinium* alkaloids are also responsible for livestock intoxication^9^ due to their action on α1 nAChRs expressed at neuromuscular junctions.^10^ However, it has been reported previously that MLA **1** has higher affinity for α7 nAChRs compared to other nAChR subtypes.^6,11,12^

**Figure 1:**
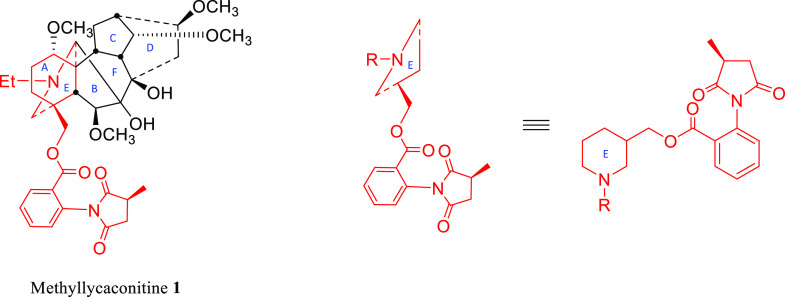
E-ring analogue system of MLA **1**.

Several structural features of MLA **1** have been studied in our ongoing structure–activity relationship (SAR) studies. For example, it was found that the nitrogen atom plays a key role in the pharmacological action of NDAs.^13^ Also, the ester side-chain is an important moiety as MLA **1** lost 1000-fold of its activity when converted to neopentyl alcohol lycoctonine.^14−16^ Furthermore, the side-chain on the nitrogen atom affects the interaction with the target nAChR. Several piperidine (ring E) analogues of MLA have been synthesized (Figure 1) with different N-side-chains (methyl, ethyl, *n*-butyl, 2-phenylethyl, 3-phenylpropyl, diethyl ether, and 2-phenylethyl ether) and tested on bovine adrenal α3β4 nAChRs, where the best analogue had a 3-phenylpropyl N-side-chain.^17^ This analogue system was tested on the α7 nAChR in a competition binding experiment on rat brain preparations using [^125^I]αBGT, where the best analogue (3-phenylpropyl N-side-chain) showed little inhibition with an IC_50_ = 177 μM, while other analogues showed no inhibition with IC_50_ > 300 μM.^18^

**Scheme 1:**
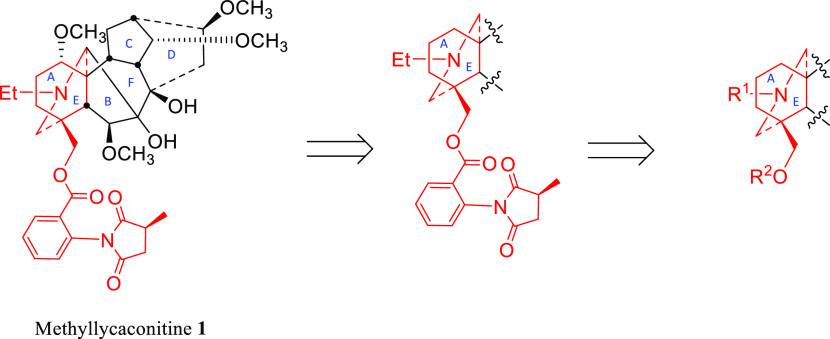
Relationship of MLA **1** to the Target AE-Bicyclic System

The aim of this study was to synthesize AE-bicyclic analogues of MLA **1** with different nitrogen and ester side-chains (Scheme 1) and to examine their ability to modulate the activity of human α7 nAChRs, with the aim of obtaining a better SAR understanding of these compounds.

## Results and Discussion

### Synthesis of the AE-Bicyclic Core

**Scheme 2:**
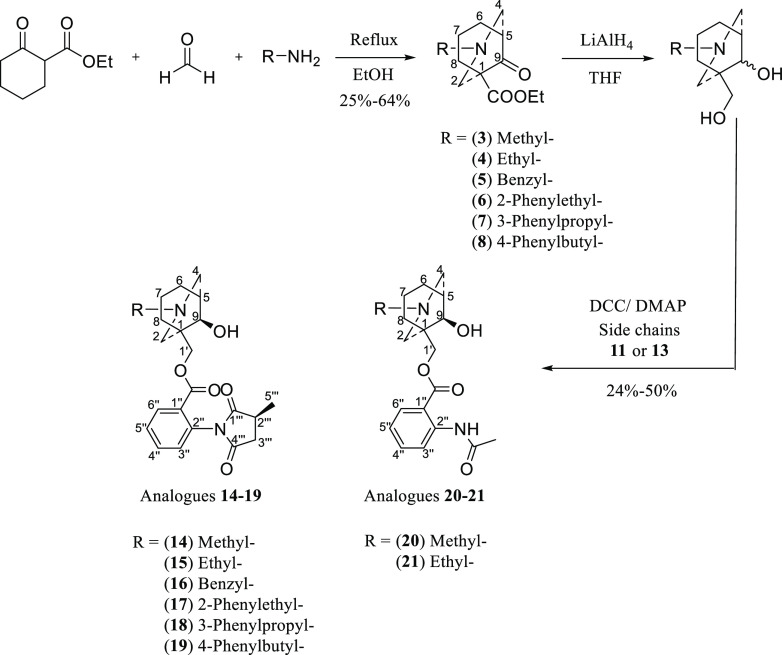
Synthesis of AE-Bicyclic Analogues **14**–**21**

The synthesis of MLA analogues starts with the core synthesis using the classical double Mannich reaction^18,19^ where different amines were used to obtain different N-side-chains. The side-chains (methyl, ethyl, benzyl, 2-phenylethyl, 3-phenylpropyl, and 4-phenylbutyl) (Scheme 2) were selected to investigate the importance of the hydrophobic interactions. The reaction was accomplished by heating the reactants under reflux in ethanol for 4 h. As the boiling point of the methylamine solution (40 wt % in water) is 48 °C, the synthesis of compound **3** was also achieved at 20 °C for 2 d with no significant drop in yield.

### Reduction of the AE-Bicyclic Core Using LiAlH_4_ (LAH)

**Figure 2:**
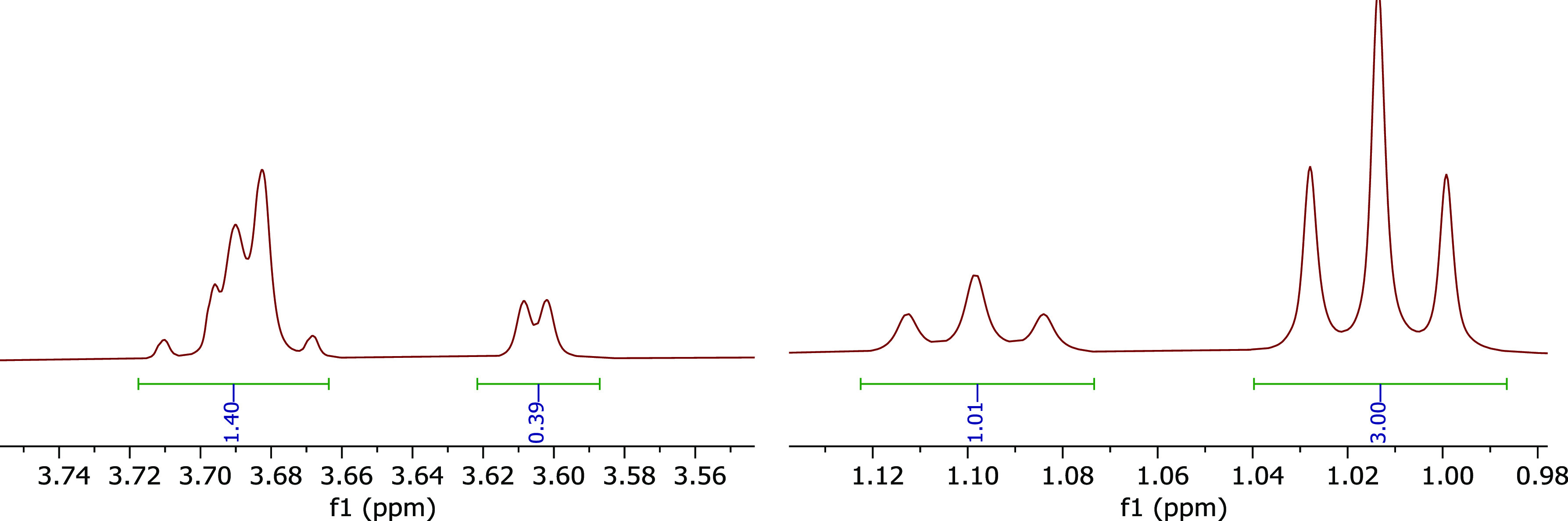
^1^H NMR (500 MHz) of 9-H proton (left) and the methyl of *N*CH_2_**CH**_**3**_ (right) of the epimeric mixture after reduction of cyclohexanone **4**.

The reduction of the AE-bicyclic compounds **3**–**8** was performed using LAH in anhydrous THF under N_2_ gas (Scheme 2), and the reaction was monitored by TLC and quenched after 7 h using the Fieser method, where *X* mL (*X* = grams of LAH) of water was added slowly followed by *X* mL of 15% aq. sodium hydroxide solution and then 3*X* mL of water. The resulting mixture was stirred with magnesium sulfate for 10 min and then filtered over Celite and evaporated to dryness. The reduction results in epimeric secondary alcohol at position 9. As an example, cyclohexanone **4** was reduced to get both epimers at position 9. The ^1^H NMR methyl triplet signals of the *N*CH_2_**CH**_**3**_ showed that the ratio of the isomers is 3:1 (Figure 2). The ^1^H NMR spectrum also shows the difference in the intensity of 9-H signals in both epimers (3.60 ppm vs 3.68 ppm) (Figure 2). The full ^1^H and ^13^C NMR spectra also showed different intensities of signals for the 3:1 isomeric ratio (SI Figure S1).

**Figure 3:**
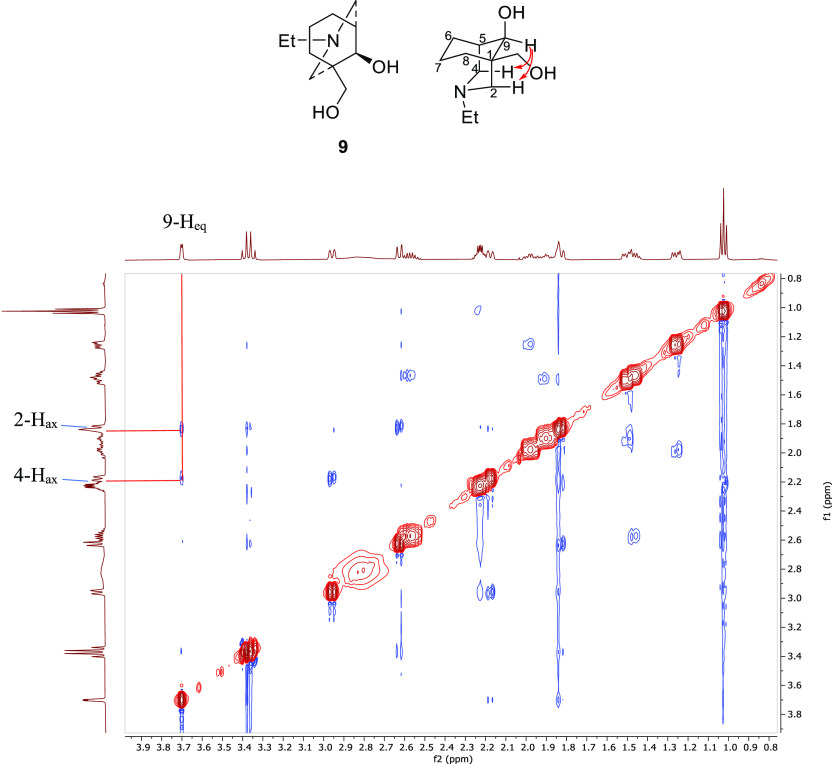
NOESY correlation between 9-H_eq_ and 2-H_ax_ and 4-H_ax_ in diol **9**.

The mixture was purified using column chromatography to obtain the major isomer, diol **9**. The β-alcohol was established by NOESY as the proton at position 9 showed a correlation with protons 2ax and 4ax (Figure 3). The methylene protons (CH_2_OH) resonate as two adjacent doublets (3.35 and 3.39 ppm) while they showed as a multiplet in the epimeric mixture (Figure 2). The compounds **3** and **5**–**8** were reduced and used in the esterification step without purification.

### Synthesis of the Carboxylic Acid Side-Chains

**Figure 4:**
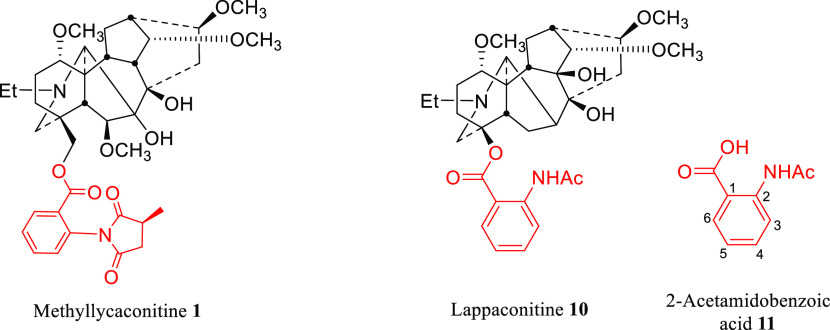
Side-chains (red) of methyllycaconitine **1** and lappaconitine **10**.

MLA **1** is a potent nAChR antagonist. Lappaconitine **10** is the most clinically successful NDA where its hydrobromide salt (Allapinin) is used as an antiarrhythmic drug.^8^ Therefore, their side-chains (Figure 4) were chosen to be attached to the analogues.

#### Synthesis of Lappaconitine Side-Chain

The synthesis of 2-acetamidobenzoic acid **11** was accomplished through refluxing anthranilic acid and acetic anhydride in anhydrous tetrahydrofuran under nitrogen gas for 4 h. The reaction was quenched using 1 M aq. HCl, and the product was recrystallized from water and ethanol (1:1).

#### Synthesis of Methyllycaconitine Side-Chain

**Scheme 3:**
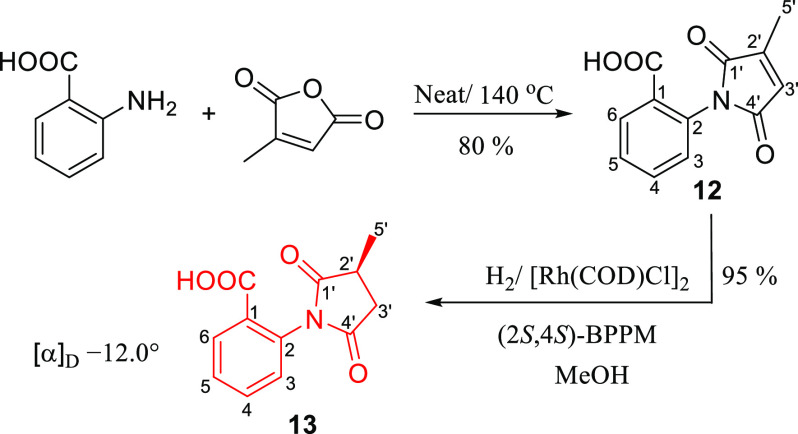
Synthesis of MLA Side-Chain

The first step of MLA **1** side-chain synthesis was performed by neat fusion of anthranilic acid and citraconic anhydride at 140 °C under nitrogen gas for 24 h (Scheme 3).^19−21^ Chiral hydrogenation of **12** to get the *S*-enantiomer was tried with (*S*)-ruthenium diacetate (2,2′-bis(diphenylphosphino)-1,1′-binaphthyl) (*S*-Ru(OAc)_2_BINAP) without success. Then it was performed using (2*S*,4*S*)-1-Boc-4-diphenylphosphino-2-(diphenylphosphinomethyl)pyrrolidine (BPPM) coupled with rhodium cyclooctadiene chloride dimer (Rh(COD)Cl)_2_.^22−24^ The optical rotation was −12.0°, consistent with the literature value.^25^

**Figure 5:**
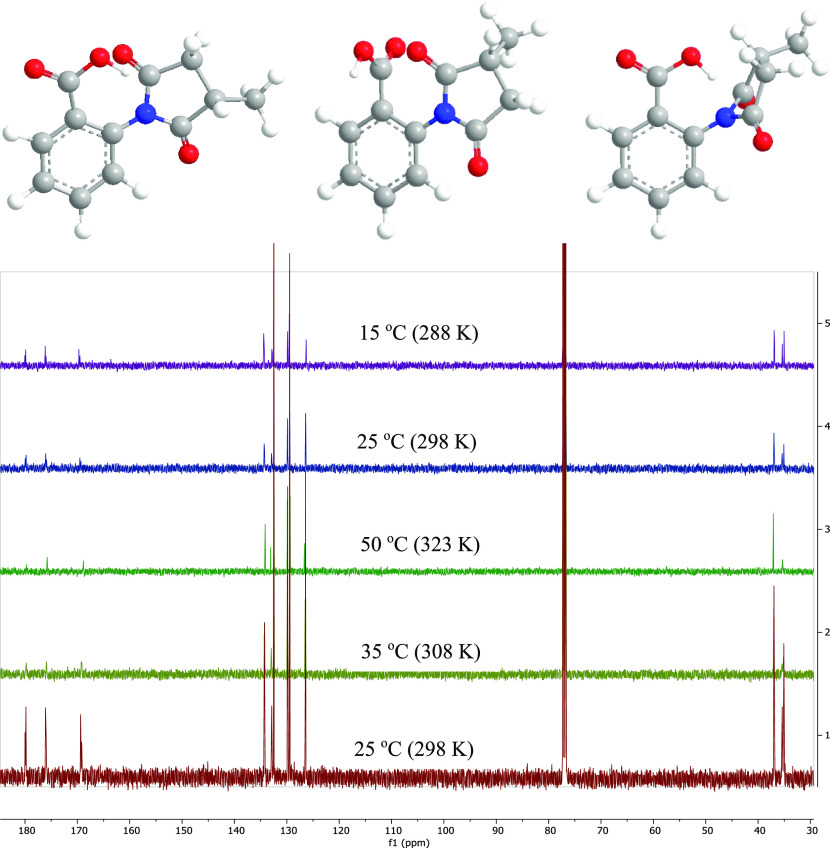
^13^C NMR (125 MHz) in CDCl_3_ of **13** at various temperatures.

The ^13^C NMR of **13** showed doubling phenomena at 25 °C when measured in CDCl_3_, which could be due to an intramolecular interaction that hindered the free rotation of the methyl succinimide group. Variable temperature (VT) NMR experiments were performed, and the doubling phenomena disappeared on increasing the temperature to 55 °C where the molecule has more energy to rotate freely, and then reappeared upon cooling down to 25 and 15 °C (Figure 5). To explain the hindrance that results in the NMR doubling, the 3D models in Figure 5 show that the clash happens between the carboxylic acid and the methylsuccinimide moiety.

NMR of **13** was also measured in CD_3_OD to check if the doubling happens due to intramolecular H-bonding or steric clash. ^13^C NMR spectra in CD_3_OD again showed doubling of the signals (SI Figure S2) consistent with steric hindrance in methylsuccinimido anthranilate **13**.

### Synthesis of the Analogues by Esterification

The reduced AE-bicycles were esterified with the naturally occurring NDA side-chains (**11** and **13**) using *N*,*N*′-dicyclohexyl-carbodiimide (DCC) and 4-dimethylaminopyridine (DMAP) in anhydrous acetonitrile at 40 °C under anhydrous nitrogen gas (Scheme 2). The reaction was monitored and stopped after 24 h, and the crude material was purified to homogeneity to yield analogues **14**–**21**.

The stereochemistry of the hydroxy group at position 9 was determined to be axial by NOESY spectrum as the 9-H_eq_ showed correlation with 2-H_ax_ and 4-H_ax_. Analogues **14** and **21** were taken as examples, and SI Figure S3 shows the NOE correlations in both of them.

The NMR spectra of the analogues were similar, the only major difference observed was for the protons at carbon 1′, which showed the roofing effect in analogues **20** and **21** with the 2-acetamido-benzoic acid side-chain where this could be caused by a steric hindrance effect from the side-chain. They merge into one multiplet signal in analogues **14**–**19** with the 2-methylsuccinimidobenzoic acid side-chain. SI Figure S4 shows the ^1^H NMR signal at position 1′ in analogues **14**–**15** and **20**–**21**.

As we have reported with some naturally occurring NDAs,^26^ these simple analogues show the effect of steric compression on the axial proton of position 7. The equatorial protons resonate usually at a higher frequency due to the anisotropic effect of the C–C bond.^27^ In these bicyclic compounds, the axial proton at position 7 is further downfield due to the interaction with the nitrogen lone pair of electrons. The chemical shift difference that was observed in the bicyclic compounds **3**–**9** and **14**–**21** is ∼1–1.5 ppm. The axial protons at position 6 and 8 also resonate at a higher chemical shift, which is probably due to 1–3 interactions through space with substituents at positions 1 and 9.^28^

### Antagonist Activity of MLA Analogues on Human α7 nAChRs

**Figure 6:**
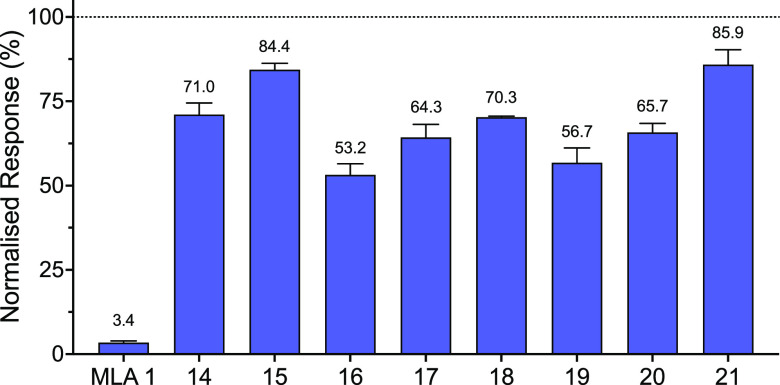
Normalized agonist (ACh) response of α7 nAChRs in the presence of MLA or analogues **14**–**21**. Data were generated from cloned human α7 nAChRs expressed in *Xenopus* oocytes. Data are mean ± SEM of at least three independent experiments.

The antagonistic activity of MLA **1** and the analogues **14**–**21** has been tested on human α7 nAChRs heterologously expressed in *Xenopus* oocytes. The level of antagonism was measured by the coapplication of the analogues [1 nM] with an EC_50_ concentration of ACh [100 μM], after a preapplication of the analogues for 2 min. Responses to ACh in the presence of MLA analogues were normalized to responses to an EC_50_ concentration of ACh [100 μM] applied in the absence of analogues (Figure 6). MLA **1** inhibited the receptor response to 3.4 ± 0.2% (*n* = 4) of normalized responses. In addition, all of the analogues exhibited antagonist effects at human α7 nAChRs, resulting in significantly reduced agonist responses (*P* < 0.0001; Figure 6). Compounds **16**, **19**, and **17** showed the highest levels of antagonism, agonist responses to 53.2 ± 1.9% (*n* = 3), 56.7 ± 2.5% (*n* = 3), and 64.3 ± 2.2% (*n* = 3) of normalized responses, respectively (Figure 6).

**Figure 7:**
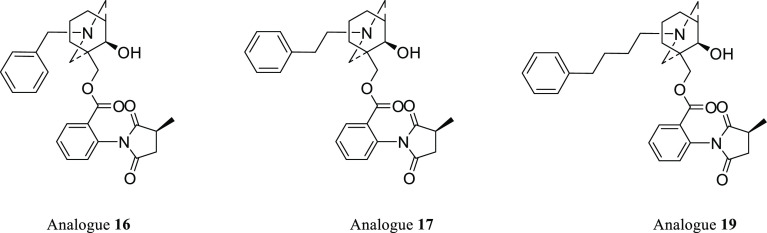
The three most active analogues, **16**, **17**, and **19**.

The antagonist activity of these analogues showed little advantage of the (*S*)-2-methylsuccinimido benzoate ester side-chain especially when comparing analogue **14** with **20**. The data for analogues **14**–**19** highlights the effect of the N-side-chain on the antagonist activity at human α7 nAChR where the activity is in the following order: benzyl > 4-phenylbutyl > 2-phenylethyl > 3-phenylpropyl > methyl > ethyl (Figure 7). These data indicate that a bulkier N-side-chain (with phenyl moiety) enhances the activity compared to alkane side-chains. The E-ring analogue system developed by Bergmeier, McKay and co-workers^17,18,29−31^ was tested on bovine adrenal α3β4 nAChRs and showed that the best analogue, 3-phenylpropyl N-side-chain, inhibits the nicotine-stimulated catecholamine secretion [50 μM] by around 86%^17^ with IC_50_ = 11.4 μM^18^ compared to 95% inhibition of the nicotine-stimulated catecholamine secretion [50 μM]^17^ with IC_50_ = 2.6 μM^18^ for MLA **1**. In addition, this analogue system was tested on α7 nAChRs in a competition binding experiment on rat brain preparations using [^125^I]αBGT where the best analogue (3-phenylpropyl N-side-chain) showed only a little inhibition with IC_50_ = 177 μM compared to 0.01 μM for MLA **1**.^18^ The AE-bicyclic analogues showed better activity compared to the reported one (E) ring system where the best analogue **16**, benzyl N-side-chain, inhibits the agonist response at human α7 nAChR to around 53% [1 nM].

## Conclusions

Several MLA **1** AE-bicyclic analogues were synthesized with different N-side-chains and ester side-chains. Antagonist effects of synthetic analogues were examined on human α7 nAChRs and compared to that of MLA **1**. The antagonist activity of these analogues showed little advantage of the (*S*)-2-methylsuccinimido benzoate ester side-chain especially when comparing analogue **14** with **20**. The data from analogues **14**–**19** highlight the effect of the N-side-chain on the antagonist activity at human α7 nAChRs, where a bulkier N-side-chain (with a phenyl moiety) enhanced the antagonist activity compared to alkane side-chains. The pharmacological results achieved with these AE-bicyclic analogues, synthesized in three steps, showed better activity compared to the reported one ring system. The best analogue **16**, containing a benzyl N-side-chain, inhibited the agonist response at human α7 nAChRs to around 53% [1 nM]. However, these are significantly less efficacious than MLA **1** so further optimization will be required to achieve comparable antagonist activity. In addition, it may be of interest to undertake further studies to examine the selectivity of these novel compounds for α7 nAChRs by examining their influence on a broader range of nAChR subtypes.

## Experimental Section

### General Methods

Analytical thin layer chromatography (TLC) was performed using aluminum backed sheet precoated silica gel plates (Merck Kieselgel 60 F254). Compounds were visualized by UV light or by staining with iodine, ninhydrin, and *p*-anisaldehyde. Column chromatography was performed over silica gel 200–400 mesh (purchased from Sigma-Aldrich).^1^H NMR spectra were recorded with a Bruker Avance III (500 MHz) spectrometer at 25 °C. Chemical shifts are given in parts per million (ppm), referenced to the residual solvent peak, and reported as position (δ), multiplicity (s = singlet, br = broad, d = doublet, dd = doublet of doublets, t = triplet, dt = doublet of triplets, tt = triplet of triplets, q = quartet, qd = quartet of doublets, qt = quartet of triplets, quin = quintet, m = multiplet), relative integral, assignment, and coupling constant (*J* in Hz). ^13^C NMR spectra were recorded with a Bruker Avance III (125 MHz) spectrometer at 25 °C with complete proton decoupling. Chemical shifts are expressed in parts per million (ppm) referenced to the used solvent, and reported as position (δ). In addition, ^1^H–^1^H COSY, ^1^H–^13^C HMBC, and ^1^H–^13^C HSQC correlation spectra were used for the complete assignment of the proton and carbon resonances. ^1^H–^1^H NOESY NMR spectra were recorded in special cases to determine the stereochemistry of diastereoisomers. High Resolution Time-of Flight (HR TOF) mass spectra (MS) were obtained on a Bruker Daltonics “micrOTOF” mass spectrometer using electrospray ionization (ESI) (loop injection +ve and −ve mode). A PerkinElmer 65 spectrum FT-IR spectrometer was used to obtain the IR spectra. Optical rotations were recorded on an Optical Activity LTD high performance polarimeter using halogen spectral line 589 nm. The final compounds tested for biological activity were all >98% pure; indeed analytical HPLC showed that the purity of **18** was 98%; all seven other analogues were >99% pure (HPLC traces for compounds **14**–**21** are provided in the SI). These compounds were also all homogeneous by TLC and NMR.

#### Ethyl 3-Methyl-9-oxo-3-azabicyclo[3.3.1]nonane-1-carboxylate (**3**)

A solution of ethyl cyclohexanone-2-carboxylate (4.44 mmol, 0.748 mL, 95%), 2.2 equiv of formaldehyde (9.768 mmol, 0.713 mL, 38% aq v/v) and 1.1 equiv of methylamine (4.88 mmol, 0.608 mL, 33% in EtOH) in ethanol (25 mL) was stirred at 40 °C for 2 d under N_2_. Then the solution was concentrated under vacuum and purified by column chromatography using 12.5% EtOAc in petroleum ether to yield the title compound **3** (280 mg, 28%) as a yellow oil. R_f_ = 0.36 (12.5% EtOAc in petroleum ether). HRMS (ESI): *m*/*z* calcd. for C_12_H_20_NO_3_: 226.1443, found: 226.1443 [M + H]^+^ and *m*/*z* calcd. for C_12_H_19_NO_3_Na: 248.1263, found: 248.1262 [M + Na]^+^. ν_max_ (NaCl)/cm^–1^ 1733 (ester, C=O), 1717 (ketone, C=O). ^1^H NMR (500 MHz, CDCl_3_): δ (ppm) = 1.26 (t, *J* = 7.1 Hz, 3H, OCH_2_**CH**_**3**_), 1.49–1.57 (m, 1H, H7_eq_), 2.00–2.09 (m, 1H, H6_eq_), 2.10–2.17 (m, 1H, H6_ax_), 2.15–2.29 (m, 1H, H8_eq_), 2.25 (s, 3H, NCH_3_), 2.40–2.45 (m, 1H, H5_eq_), 2.50 (dddd, *J* = 14.2, 12.3, 6.3, 2.0 Hz, 1H, H8_ax_), 2.59 (dd, *J* = 11.2, 3.8 Hz, 1H, H4_ax_), 2.76–2.89 (m, 1H, H7_ax_), 2.96 (dd, *J* = 11.3, 1.9 Hz, 1H, H2_ax_), 3.04 (dt, *J* = 11.2, 2.3 Hz, 1H, H4_eq_), 3.11 (dd, *J* = 11.5, 2.2 Hz, 1H, H2_eq_), 4.19 (q, *J* = 7.1 Hz, 2H, O**CH**_**2**_CH_3_). ^13^C NMR (125 MHz, CDCl_3_): δ (ppm) = 13.8 (OCH_2_**CH**_**3**_), 20.2 (C7), 34.0 (C6), 36.8 (C8), 44.8 (N CH_3_), 47.1 (C5), 58.5 (C1), 60.9 (O**CH**_**2**_CH_3_), 62.3 (C4), 64.0 (C2), 170.9 (ester), 212.3 (C9).

#### Ethyl 3-Ethyl-9-oxo-3-azabicyclo[3.3.1]nonane-1-carboxylate (**4**)

A solution of ethyl cyclohexanone-2-carboxylate (25.07 mmol, 4.09 mL, 98%), 2.2 equiv of formaldehyde (55.154 mmol, 2.19 mL, 38% aq v/v), and 1.1 equiv of ethylamine (27.577 mmol, 3.99 mL, 70% aq v/v) in ethanol (170 mL) was heated under reflux for 3 h under N_2_. Then the solution was cooled and concentrated under vacuum, and purified by column chromatography using 10% EtOAc in petroleum ether to yield the title compound **4** (3.75 g, 63%) as a yellow oil. R_f_ = 0.25 (10% EtOAc in petroleum ether). HRMS (ESI): *m*/*z* calcd. for C_13_H_22_NO_3_: 240.1600, found: 240.1600 [M + H]^+^ and *m*/*z* calcd. for C_13_H_21_NO_3_Na: 262.1419, found: 262.1418 [M + Na]^+^. ν_max_ (NaCl)/cm^–1^ 1733 (ester, C=O), 1716 (ketone, C=O). ^1^H NMR (500 MHz, CDCl_3_): δ (ppm) = 1.10 (t, *J* = 7.2 Hz, 3H, NCH_2_**CH**_**3**_), 1.28 (t, *J* = 7.1 Hz, 3H, OCH_2_**CH**_**3**_), 1.46–1.57 (m, 1H, H7_eq_), 2.00–2.18 (m, 2H, H6_ax_ and H6_eq_), 2.19–2.28 (m, 1H, H8_eq_), 2.37–2.60 (m, 5H, N**CH**_**2**_CH_3,_ H5, H8_ax_ and H4_ax_), 2.78–2.90 (m, 1H, H7_ax_), 2.94 (d, *J* = 12.0 Hz, 1H, H2_ax_), 3.15 (d, *J* = 11.1 Hz, 1H, H4_eq_), 3.22 (d, *J* = 11.4 Hz, 1H, H2_eq_), 4.21 (q, *J* = 7.1 Hz, 2H, O**CH**_**2**_CH_3_). ^13^C NMR (125 MHz, CDCl_3_): δ (ppm) = 12.7 (NCH_2_**CH**_**3**_), 14.1 (OCH_2_**CH**_**3**_), 20.5 (C7), 34.1 (C6), 36.8 (C8), 47.2 (C5), 51.1 (N**CH**_**2**_CH_3_), 58.8 (C1), 59.9 (C4), 61.0 (O**CH**_**2**_CH_3_), 61.6 (C2), 171.1 (ester), 212.6 (C9).

#### Ethyl 3-Benzyl-9-oxo-3-azabicyclo[3.3.1]nonane-1-carboxylate (**5**)

A solution of ethyl cyclohexanone-2-carboxylate (9.95 mmol, 1.62 mL, 98%), 2.2 equiv of formaldehyde (21.89 mmol, 1.6 mL, 38% aq v/v), and 1.1 equiv of benzylamine (10.9 mmol, 1.2 mL, 99%) in ethanol (70 mL) was heated under reflux for 3 h under N_2_. Then the solution was cooled and concentrated under vacuum, and purified by column chromatography using 10% EtOAc in petroleum ether to yield the title compound **5** (750 mg, 25%) as a yellow oil. R_f_ = 0.29 (10% EtOAc in petroleum ether). HRMS (ESI): *m*/*z* calcd. for C_18_H_24_NO_3_: 302.1756, found: 302.1755 [M + H]^+^ and *m*/*z* calcd. for C_18_H_23_NO_3_Na: 324.1576, found: 324.1573 [M + Na]^+^. ν_max_ (NaCl)/cm^–1^ 1732 (ester, C=O), 1717 (ketone, C=O). ^1^H NMR (500 MHz, CDCl_3_): δ (ppm) = 1.27 (t, *J* = 7.1 Hz, 3H, OCH_2_**CH**_**3**_), 1.55- 1.65 (m, 1H, H7_eq_), 2.02–2.19 (m, 2H, H6_ax_ and H6_eq_), 2.20–2.27 (m, 1H, H8_eq_), 2.44–2.48 (m, 5H), 2.54 (dddd, *J* = 14.1, 12.2, 6.4, 1.9 Hz, 1H, H8_ax_), 2.63 (dd, *J* = 10.9, 2.5 Hz, 1H, H4_ax_), 2.92–3.06 (m, 2H, H2_ax_ and H7_ax_), 3.13 (d, *J* = 11.2, 2.4 Hz, 1H, H4_eq_), 3.20 (dd, *J* = 11.5, 2.4 Hz, 1 H, H2_eq_), 3.52 (s, 2H, N**CH**_**2**_Ph), 4.19 (qd, *J* = 7.2, 3.0 Hz, 2H, O**CH**_**2**_CH_3_), 7.27–7.36 (m, 5H, NCH_2_**Ph**). ^13^C NMR (125 MHz, CDCl_3_): δ (ppm) = 14.1 (OCH_2_**CH**_**3**_), 20.7 (C7), 34.1 (C6), 36.7 (C8), 47.2 (C5), 58.9 (C1), 60.3 (C4), 61.1 (O**CH**_**2**_CH_3_), 61.8 (C2), 62.1 (N**CH**_**2**_Ph), 127.2 (C4 arom), 128.4, 128.7 (C2 arom, C3 arom, C5 arom and C6 arom), 138.3 (C1 arom), 170.9 (ester), 212.4 (C9).

#### Ethyl 3-(2-Phenylethyl)-9-oxo-3-azabicyclo[3.3.1]nonane-1-carboxylate (**6**)

A solution of ethyl cyclohexanone-2-carboxylate (3.17 mmol, 0.517 mL, 98%), 2.2 equiv of formaldehyde (7 mmol, 0.525 mL, 38% aq. v/v), and 1.1 equiv of 2-phenylethyl amine (3.49 mmol, 0.446 mL, 99%) in ethanol (20 mL) was heated under reflux for 3 h under N_2_. Then the solution was cooled and concentrated under vacuum and purified by column chromatography using 10% EtOAc in petroleum ether to yield the title compound **6** (640 mg, 64%) as a yellow oil. R_f_ = 0.30 (10% EtOAc in petroleum ether). HRMS (ESI): *m*/*z* calcd. for C_19_H_26_NO_3_: 316.1913, found: 316.1912 [M + H]^+^ and *m*/*z* calcd. for C_19_H_25_NO_3_Na: 338.1732, found: 338.1731 [M + Na]^+^. ν_max_ (NaCl)/cm^–1^ 1732 (ester, C=O), 1716 (ketone, C=O). ^1^H NMR (500 MHz, CDCl_3_): δ (ppm) = 1.28 (t, *J* = 7.1 Hz, 3H, OCH_2_**CH**_**3**_), 1.37–1.45 (m, 1H, H7_eq_), 1.97–2.11 (m, 2H, H6_ax_ and H6_eq_), 2.12–2.20 (m, 1H, H8_eq_), 2.42–2.53 (m, H5 and H8_ax_), 2.55–2.68 (m, 4H, H7_ax_, H4_ax_ and N**CH**_**2**_CH_2_Ph), 2.82 (t, *J* = 7.5 Hz, 2H, NCH_2_**CH**_**2**_Ph), 3.02 (d, *J* = 11.3 Hz, 1H, H2_ax_), 3.18 (d, *J* = 11.0 Hz, 1H, H4_eq_), 3.27 (d, *J* = 11.4 Hz, 1 H, H2_eq_), 4.21 (q, *J* = 7.1 Hz, 2H, O**CH**_**2**_CH_3_), 7.18–7.32 (m, 5H, NCH_2_CH_2_**Ph**). ^13^C NMR (125 MHz, CDCl_3_): δ (ppm) = 14.1 (OCH_2_**CH**_**3**_), 20.2 (C7), 33.8 (NCH_2_**CH**_**2**_Ph), 34.1 (C6), 36.8 (C8), 47.2 (C5), 58.5 (N**CH**_**2**_CH_2_Ph), 58.8 (C1), 60.2 (C4), 61.1 (O**CH**_**2**_CH_3_), 61.8 (C2), 126.0 (C4 arom), 128.3 (C2 arom and C6 arom), 128.6 (C3 arom and C5 arom), 140.1 (C1 arom), 171.1 (ester), 212.5 (C9).

#### Ethyl 3-(3-Phenylpropyl)-9-oxo-3-azabicyclo[3.3.1]nonane-1-carboxylate (**7**)

A solution of ethyl cyclohexanone-2-carboxylate (3 mmol, 0.49 mL, 99%), 2.2 equiv of formaldehyde (6.6 mmol, 0.48 mL, 38% aq. v/v), and 1.1 equiv of 3-phenylpropyl amine (3.3 mmol, 0.48 mL, 99%) in ethanol (20 mL) was heated under reflux for 3 h under N_2_. Then the solution was concentrated under vacuum and purified by column chromatography using 10% EtOAc in petroleum ether to yield the title compound **7** (540 mg, 54%) as a yellow oil. R_f_ = 0.34 (10% EtOAc in petroleum ether). HRMS (ESI): *m*/*z* calcd. for C_20_H_28_NO_3_: 330.2069, found: 330.2068 [M + H]^+^ and *m*/*z* calcd. for C_20_H_27_NO_3_Na: 352.1889, found: 352.1887 [M + Na]^+^. ν_max_ (NaCl)/cm^–1^ 1732 (ester, C=O), 1716 (ketone, C=O). ^1^H NMR (500 MHz, CDCl_3_): δ (ppm) = 1.28 (t, *J* = 7.1 Hz, 3H, OCH_2_**CH**_**3**_), 1.52- 1.62 (m, 1H, H7_eq_), 1.83 (quin, *J* = 7.2 Hz, 1H, NCH_2_**CH**_**2**_CH_2_Ph), 2.05–2.16 (m, 2H, H6_ax_ and H6_eq_), 2.21–2.29 (m, 1H, H8_eq_), 2.36 (t, *J* = 7.0 Hz, 2H, N**CH**_**2**_CH_2_CH_2_Ph), 2.45–2.49 (m, 1H, H5), 2.51–2.61 (m, 2H, H4_ax_ and H8_ax_), 2.7 (t, *J* = 7.7 Hz, 2H, NCH_2_CH_2_**CH**_**2**_Ph), 2.84–2.97 (m, 2H, H2_ax_ and H7_ax_), 3.15 (dt, *J* = 11.1, 2.4 Hz, 1H, H4_eq_), 3.21 (dd, *J* = 11.4, 2.3 Hz, 1 H, H2_eq_), 4.21 (q, *J* = 7.1 Hz, 2H, O**CH**_**2**_CH_3_), 7.16–7.22, 7.26–7.32 (m, 5H, NCH_2_CH_2_CH_2_**Ph**). ^13^C NMR (125 MHz, CDCl_3_): δ (ppm) = 14.1 (OCH_2_**CH**_**3**_), 20.6 (C7), 29.1 (N CH_2_**CH**_**2**_CH_2_Ph), 33.5 (NCH_2_CH_2_**CH**_**2**_Ph) 34.2 (C6), 36.8 (C8), 47.2 (C5), 56.4 (N**CH**_**2**_CH_2_CH_2_Ph), 58.8 (C1), 60.4 (C4), 61.1 (O**CH**_**2**_CH_3_), 62.0 (C2), 125.8 (C4 arom), 128.37, 128.40 (C2 arom, C6 arom, C3 arom and C5 arom), 142.0 (C1 arom), 171.1 (ester), 212.5 (C9).

#### Ethyl 3-(4-Phenylbutyl)-9-oxo-3-azabicyclo[3.3.1]nonane-1-carboxylate (**8**)

A solution of ethyl cyclohexanone-2-carboxylate (2.91 mmol, 0.476 mL, 95%), 2.2 equiv of formaldehyde (5.83 mmol, 0.425 mL, 38% aq v/v), and 1.1 equiv of 4-phenylbutylamine (3.205 mmol, 0.52 mL, 98%) in ethanol (20 mL) was heated under reflux for 3 h under N_2_. Then the solution was cooled and concentrated under vacuum and purified by column chromatography using 10% EtOAc in petroleum ether to yield the title compound **8** (600 mg, 60%) as a yellow oil. R_f_ = 0.35 (10% EtOAc in petroleum ether). HRMS (ESI): *m*/*z* calcd. for C_21_H_30_NO_3_: 344.2226, found: 344.2227 [M + H]^+^ and *m*/*z* calcd. for C_21_H_29_NO_3_Na: 366.2045, found: 366.2044 [M + Na]^+^. ν_max_ (NaCl)/cm^–1^ 1733 (ester, C=O), 1717 (ketone, C=O). ^1^H NMR (500 MHz, CDCl_3_): δ (ppm) = 1.28 (t, *J* = 7.2 Hz, 3H, OCH_2_**CH**_**3**_), 1.50- 1.68 (m, 3H, H7_eq_ and NCH_2_**CH**_**2**_CH_2_CH_2_Ph), 1.66–1.74 (m, 2H, NCH_2_CH_2_**CH**_**2**_CH_2_Ph), 2.02–2.16 (m, 2H, H6_ax_ and H6_eq_), 2.19–2.25 (m, 1H, H8_eq_), 2.35 (td, *J* = 7.0, 1.4 Hz, 2H, N**CH**_**2**_CH_2_CH_2_CH_2_Ph), 2.42–2.46 (m, 1H, H5), 2.49–2.57 (m, 2H, H4_ax_ and H8_ax_), 2.65 (t, *J* = 7.6 Hz, 2H, NCH_2_CH_2_CH_2_**CH**_**2**_Ph), 2.80–2.88 (m, 1H, H7_ax_), 2.91 (dd, *J* = 11.5, 2.0 Hz, 1H, H2_ax_) 3.10 (dt, *J* = 11.2, 2.4 Hz, 1 H, H4_eq_), 3.17 (dd, *J* = 11.4, 2.3 Hz, 1H, H2_eq_), 4.20 (q, *J* = 7.1 Hz, 2H, O**CH**_**2**_CH_3_), 7.16–7.21, 7.26–7.31 (m, 5H, NCH_2_CH_2_CH_2_CH_2_**Ph**). ^13^C NMR (125 MHz, CDCl_3_): δ (ppm) = 14.1 (OCH_2_**CH**_**3**_), 20.5 (C7), 26.7 (NCH_2_**CH**_**2**_CH_2_CH_2_Ph), 29.0 (NCH_2_CH_2_**CH**_**2**_CH_2_Ph), 34.1 (C6), 35.6 (NCH_2_CH_2_CH_2_**CH**_**2**_Ph), 36.8 (C8), 47.2 (C5), 56.8 (N**CH**_**2**_CH_2_CH_2_CH_2_Ph), 58.8 (C1), 60.4 (C4), 61.0 (O**CH**_**2**_CH_3_), 62.0 (C2), 125.7 (C4 arom), 128.26 (C2 arom and C6 arom), 128.31 (C3 arom and C5 arom), 142.4 (C1 arom), 171.1 (ester), 212.6 (C9).

#### (9*R*)-3-Ethyl-1-hydroxymethyl-3-azabicyclo[3.3.1]nonan-9-ol (**9**)

LiAlH_4_ (1.756 mmol, 66.6 mg) was added to a solution of cyclohexanone **4** (0.878 mmol, 210 mg) (which was dried under high vacuum for 24 h) in anhydrous THF (5 mL), and the reaction stirred for 7 h at 19 °C under N_2_. Then the mixture was quenched with 66 μL of water, followed by 66 μL of sodium hydroxide solution (15%w/v) and then 200 μL water. The resulting mixture was stirred with anhydrous magnesium sulfate for 15 min and filtered over Celite. The filtrate was concentrated under vacuum and purified over column chromatography with 5–20% MeOH in DCM to yield the title compound **9** (60 mg, 34%) as a yellow oil. R_f_ = 0.27 (20% MeOH in DCM). HRMS (ESI): *m*/*z* calcd. for C_11_H_22_NO_2_: 200.1651, found: 200.1648 [M + H]^+^ and *m*/*z* calcd. for C_11_H_21_NO_2_Na: 222.1470, found: 222.1488 [M + Na]^+^. ν_max_ (NaCl)/cm^–1^ 3413 (OH). ^1^H NMR (500 MHz, CDCl_3_): δ (ppm) = 1.03 (t, *J* = 7.2 Hz, 3H, NCH_2_**CH**_**3**_), 1.26 (dd, *J* = 13.0, 5.6 Hz, 1H, H8_eq_), 1.43–1.54 (m, 2H, H7_eq_ and H6_eq_), 1.80–2.02 (m, 4H, H8_ax_, H6_ax_, H5 and H2_ax_), 2.18 (dt, *J* = 11.1, 2.4 Hz, 1H, H4_ax_), 2.20–2.28 (m, 2H, N**CH**_**2**_CH_3_**)**, 2.52–2.61 (m, 1H, H7_ax_), 2.63 (dd, *J* = 11.0, 1.5 Hz, 1H, H2_eq_), 2.66–2.93 (br, 2H, 9-**OH** and 1 × **OH**), 2.96 (dt, *J* = 11.1, 2.1 Hz, 1H, H4_eq_), 3.35 (d, *J* = 10.8, 1H, C**Ha**HbOH), 3.39 (d, *J* = 10.8, 1H, CHa**Hb**OH), 3.70 (d, *J* = 3.6 Hz, 1H, 9-H). ^13^C NMR (125 MHz, CDCl_3_): δ (ppm) = 12.7 (NCH_2_**CH**_**3**_), 20.5 (C7), 23.9 (C6), 26.5 (C8), 36.1 (C5), 38.1 (C1), 52.3 (N**CH**_**2**_CH_3_), 58.3 (C4), 60.4 (C2), 70.9 (C1), 75.1 (C9).

#### 2-Acetamidobenzoic Acid (**11**)

Anthranilic acid (28 mmol, 3.92 g, 98%) was heated under reflux with 5 equiv of acetic anhydride (140 mmol, 13.23 mL) and 1 equiv of anhydrous triethylamine (28 mmol, 3.94 mL, 99%) in THF (20 mL) under nitrogen for 4 h. The reaction mixture was cooled to 19 °C and then in an ice bath, then 20 mL of 1 M aq. HCl was added gradually while the reaction mixture was on ice. The precipitate was filtered and washed with ice-cold water. The product was recrystallized from water and ethanol to yield the title compound **11** (4.2 g, 84%) as pale brown crystals. R_f_ = 0.42 (10% MeOH in DCM). HRMS (ESI): *m*/*z* calcd. for C_9_H_8_NO_3_: 178.0504, found: 178.0505 [M – H]^−^ and *m*/*z* calcd. for C_10_H_10_NO_5_: 224.0559, found: 224.0606 [M + HCOO]^−^. ^1^H NMR (500 MHz, CD_3_OD): δ (ppm) = 2.18 (s, 3H, CO**CH**_**3**_), 7.11 (td, *J* = 7.7, 1.2 Hz, 1H, H5 arom), 7.52 (ddd, *J* = 8.7, 7.7, 1.7 Hz, 1H, H4 arom), 8.05 (dd, *J* = 7.7, 1.7 Hz, 1H, H6 arom), 8.52 (d, *J* = 8.4 Hz, 1H, H3 arom). ^13^C NMR (125 MHz, CD_3_OD): δ (ppm) = 25.1 (CO**CH**_**3**_), 117.4 (C1 arom), 121.4 (C3 arom), 123.9 (C5 arom), 132.5 (C6 arom), 135.1 (C4 arom), 142.3 (C2 arom), 171.24 (COOH), 171.39 (NH**C**OCH_3_).

#### 2-(3-Methyl-2,5-dioxo-2,5-dihydro-1*H*-pyrrol-1-yl)benzoic Acid (**12**)

Neat anthranilic acid (21.6 mmol, 3.02 g, 98%) was stirred with 1 equiv of citraconic anhydride (21.6 mmol, 1.98 mL, 98%) at 140 °C for 24 h under nitrogen then cooled to 19 °C. After that, the crude mixture was dissolved in EtOAc (30 mL). The organic layer was washed sequentially with 1 M HCl (2 × 20 mL), water (1 × 20 mL), and brine (1 × 20 mL). The organic layer was dried (MgSO_4_) and filtered, and the filtrate was concentrated under vacuum and purified over column chromatography with 10% MeOH in DCM to yield the title compound **12** (4.0 g, 80%) as a brownish yellow powder. R_f_ = 0.32 (10% MeOH in DCM). HRMS (ESI): *m*/*z* calcd. for C_12_H_8_NO_4_: 230.0453, found: 230.0458 [M – H]^−^ and *m*/*z* calcd. for C_13_H_10_NO_6_: 276.0508, found: 276.0528 [M + HCOO]^−^. ^1^H NMR (500 MHz, CDCl_3_): δ (ppm) = 2.18 (d, *J* = 1.7 Hz, 3H, 5′ CH_3_), 6.51 (d, *J* = 1.9 Hz, 1H, H3′), 7.32 (dd, *J* = 7.9, 1.2 Hz, 1H, H3 arom), 7.52 (td, *J* = 7.7, 1.2 Hz, 1H, H5 arom), 7.69 (td, *J* = 7.7, 1.6 Hz, 1H, H4 arom), 8.16 (dd, *J* = 7.9, 1.5 Hz, 1H, H6 arom). ^13^C NMR (125 MHz, CDCl_3_): δ (ppm) = 11.3 (5′, **C**H_3_), 127.20 (C1 arom), 128.05 (C3′), 129.15 (C5 arom), 130.54 (C3 arom), 132.16 (C2 arom), 132.44 (C6 arom), 134.25 (C4 arom), 146.4 (C2′), 169.84 (COOH), 170.20 (C4′), 170.87 (C1′).

#### (*S*)-2-(3-Methyl-2,5-dioxopyrrolidin-1-yl)benzoic Acid (**13**)

(2*S*,4*S*)-1-Boc-4-diphenylphosphino-2-(diphenylphosphinomethyl)pyrrolidine (BPPM) (0.649 mmol (5 mol %), 359 mg) and rhodium cyclooctadiene chloride dimer (Rh(COD)Cl)_2_ (0.649 mmol (5 mol %), 326 mg, 98%) were stirred together in anhydrous toluene (10 mL) under nitrogen gas for 30 min. Then the flask was vacuumed, and hydrogen was introduced. Compound **12** (0.01298 mol, 3 g) was dissolved in anhydrous methanol (10 mL) and added to the mixture. The reaction was monitored by TLC and stopped after 24 h. The mixture was concentrated under vacuum and purified over column chromatography with 10% MeOH in DCM to yield the title compound **13** (2.9 g, 95%) as a brownish yellow powder. R_f_ = 0.31 (10% MeOH in DCM). [α]_D_ −12.0° (*c* 1.0, CHCl_3_). HRMS (ESI): *m*/*z* calcd. for C_12_H_10_NO_4_: 232.0610, found: 232.0613 [M – H]^−^. ^1^H NMR (500 MHz, CDCl_3_): δ (ppm) = 1.44 (d, *J* = 6.8 Hz, 3H, 5′ CH_3_), 2.53 (d, *J* = 17.9 Hz, 1H, H3′A), 3.01–3.16 (m, 2H, H2′ and H3′ B), 7.27 (d, *J* = 7.7 Hz, 1H, H3 arom), 7.54 (t, *J* = 7.7 Hz,1H, H5 arom), 7.70 (t, *J* = 7.7 Hz, 1H, H4 arom), 8.19 (d, *J* = 7.7 Hz, 1H, H6 arom). ^13^C NMR (125 MHz, CDCl_3_): δ (ppm) = 16.5 (5′), 35.13 and 35.5 (3′), 37.0 (2′), 125.5 (C1 arom), 129.5 (C5 arom), 129.9 (C3 arom), 132.5 (C6 arom), 132.9 (C2 arom), 134.4 (C4 arom), 146.4 (C2′), 169.2 and 169.4 (COOH), 176.0 and 176.1 (C4′), 179.9 and 180.0 (C1′).

#### ((9*R*)-9-Hydroxy-3-methyl-3-azabicyclo[3.3.1]nonan-1-yl)methyl 2-((*S*)-3-Methyl-2,5-dioxopyrrolidin-1-yl)benzoate (**14**)

Compound **3** (0.89 mmol, 200 mg) was reduced using LAH (1.78 mmol, 84.2 mg) as described for compound **9**. The crude product was used for the esterification step without purification. Compound **13** (0.189 mmol, 44 mg) was stirred with DCC (0.189 mmol, 39.4 mg, 99%) and DMAP (0.0189 mmol, 2.3 mg, 99%) in anhydrous acetonitrile under nitrogen gas at 40 °C for 20 min, and then the crude amino alcohol (35 mg) was added. The reaction was monitored by TLC and stopped after 24 h. The mixture was concentrated under vacuum and purified over column chromatography with 5% MeOH in DCM to yield the title compound **14** (18 mg, 24%) as a yellow oil. R_f_ = 0.4 (5% MeOH in DCM). HRMS (ESI): *m*/*z* calcd. for C_22_H_29_N_2_O_5_: 401.2077, found: 401.2073 [M + H]^+^. ^1^H NMR (500 MHz, CD_3_OD): δ (ppm) = 1.41 (d, *J* = 6.7 Hz, 3H, 5‴), 1.44–1.56 (m, 3H, H6_eq_, H7_eq_, H8_eq_), 1.72–1.81 (m, 1H, H8_ax_), 1.86 (br s, 1H, H5), 1.99–2.07 (m, 1H, H6_ax_), 2.14–2.21 (m, 4H, N–CH_3_, H2_ax_), 2.34 (d, *J* = 11.4,1H, H4_ax_), 2.45–2.63 (m, 2H, H7_ax_, H3‴A), 2.85–2.91 (m, 1H, H2_eq_), 2.96 (d, *J* = 11.4 Hz, 1H, H4_eq_), 3.04–3.15 (m, 2H, H2‴ and H3‴B), 3.59–3.66 (m, 1H, H9), 3.96–4.11 (m, 2H, H1′ A and B), 7.35 (d, *J* = 7.4 Hz, 1H, H3″), 7.61 (d, *J* = 7.4 Hz, 1H, H5″), 7.73 (d, *J* = 7.4 Hz, 1H, H4″), 8.12 (d, *J* = 7.4 Hz, 1H, H6″). ^13^C NMR (125 MHz, CDCl_3_): δ (ppm) = 16.2 (5‴), 21.5 (C7), 25.0 (C6), 28.0 (C8), 36.18 (C2‴), 37.33 (C5), 38.00 (C3‴), 39.51 (C1), 46.4 (NCH_3_), 62.30 (C4), 64.44 (C2), 71.78 (C9), 71.80 (C1′), 128.99 (C1″), 130.42 (C5″), 131.10 (C3″), 132.07 (C6″), 133.72 (C2″), 134.50 (C4″), 165.6 (ester), 173.1 (C4‴), 181.8 (C1‴).

#### ((9*R*)-3-Ethyl-9-hydroxy-3-azabicyclo[3.3.1]nonan-1-yl)methyl 2-((*S*)-3-methyl-2,5-dioxopyrrolidin-1-yl)benzoate (**15**)

Compound **13** (0.778 mmol, 181 mg) was stirred with DCC (0.778 mmol, 162 mg, 99%) and DMAP (0.0778 mmol, 9.6 mg, 99%) in anhydrous acetonitrile under nitrogen gas at 40 °C for 20 min, and then compound **9** (155 mg) was added. The reaction was monitored by TLC and stopped after 24 h. The mixture was concentrated under vacuum and purified over column chromatography with 5% MeOH in DCM to yield the title compound **15** (130 mg, 40%) as a yellow oil. R_f_ = 0.44 (5% MeOH in DCM). HRMS (ESI): *m*/*z* calcd. for C_23_H_31_N_2_O_5_: 415.2233, found: 415.2233 [M + H]^+^. ^1^H NMR (500 MHz, CD_3_OD): δ (ppm) = 1.19 (m, 3H, NCH_2_**CH**_**3**_), 1.41 (d, *J* = 7.0 Hz, 3H, 5‴), 1.44–1.55 (m, 3H, H6_eq_, H7_eq_, H8_eq_), 1.66–1.74 (m, 1H, H8_ax_), 1.80–1.87 (m, 1H, H5), 1.97–2.05 (m, 1H, H6_ax_), 2.07–2.12 (m, 1H, H2_ax_), 2.14–2.29 (m, 3H, H4_ax_ and N**CH**_**2**_CH_3_), 2.46–2.57 (m, 1H, H3‴A), 2.58–2.69 (m, 1H, H7_ax_), 2.89–2.96 (m, 1H, H2_eq_), 3.00–3.06 (m,1H, H4_eq_), 3.05–3.14 (m, 2H, H2‴ and H3‴B), 3.76 (br s, 1H, H9), 4.00–4.16 (m, 2H, H1′ A and B), 7.36 (d, *J* = 7.8 Hz, 1H, H3″), 7.61 (d, *J* = 7.8 Hz, 1H, H5″), 7.74 (d, *J* = 7.8 Hz, 1H, H4″), 8.13 (d, *J* = 7.8 Hz, 1H, H6″). ^13^C NMR (125 MHz, CDCl_3_): δ (ppm) = 11.3 (NCH_2_**CH**_**3**_), 15.8 (5‴), 21.3 (C7), 24.8 (C6), 27.9 (C8), 35.7 (C2‴), 37.18 (C5), 37.67 (C3‴), 39.5 (C1), 53.1 (N**CH**_**2**_CH_3_), 59.4 (C4), 61.7 (C2), 70.7 (C1′), 72.0 (C9), 128.79 (C1″), 130.50 (C5″), 131.21 (C3″), 132.10 (C6″), 133.82 (C2″), 134.62 (C4″), 165.4 (ester), 175.5 (C4‴), 182.1 (C1‴).

#### ((9*R*)-3-Benzyl-9-hydroxy-3-azabicyclo[3.3.1]nonan-1-yl)methyl 2-((*S*)-3-Methyl-2,5-dioxopyrrolidin-1-yl)benzoate (**16**)

Compound **5** (1.66 mmol, 500 mg) was reduced using LAH (4.15 mmol, 157.5 mg) as described for compound **9**. The crude product was used for the esterification step without purification. Compound **13** (0.593 mmol, 138 mg) was stirred with DCC (0.593 mmol, 123.6 mg, 99%) and DMAP (0.0593 mmol, 7.3 mg, 99%) in anhydrous acetonitrile under nitrogen gas at 40 °C for 20 min, and then the crude amino alcohol (155 mg) was added. The reaction was monitored by TLC and stopped after 24 h. The mixture was concentrated under vacuum and purified over column chromatography with 5% MeOH in DCM to yield the title compound **16** (140 mg, 50%) as a yellow oil. R_f_ = 0.48 (5% MeOH in DCM). HRMS (ESI): *m*/*z* calcd. for C_28_H_33_N_2_O_5_: 477.2390, found: 477.2385 [M + H]^+^. ^1^H NMR (500 MHz, CD_3_OD): δ (ppm) = 1.37–1.43 (m, 3H, 5‴), 1.42–1.53 (m, 3H, H6_eq_, H7_eq_, H8_eq_), 1.67–1.81 (m, 1H, H8_ax_), 1.82–1.86 (m, 1H, H5), 1.95–2.06 (m, 1H, H6_ax_), 2.08–2.15 (m, 1H, H2_ax_), 2.21–2.30 (m,1H, H4_ax_), 2.43–2.63 (m, 1H, H3‴A), 2.72–2.83 (m, 1H, H7_ax_), 2.83–2.91 (m, 1H, H2_eq_), 2.90–2.96 (m,1H, H4_eq_), 3.01–3.14 (m, 2H, H2‴ and H3‴B), 3.34–3.41 (m, 2H, N**CH**_**2**_Ph), 3.60–3.68 (m, 1H, H9), 3.95–4.13 (m, 2H, H1′ A and B), 7.18–7.30 (m, 5H, NCH_2_**Ph**), 7.31–7.35 (m, 1H, H3″), 7.56 (td, *J* = 7.7, 1.3 Hz, 1H, H5″), 7.72 (td, *J* = 7.7, 1.5 Hz, 1H, H4″), 7.99 (dd, *J* = 7.7, 1.6 Hz, 1H, H6″). ^13^C NMR (125 MHz, CDCl_3_): δ (ppm) = 16.4 (5‴), 22.1 (C7), 25.1 (C6), 28.2 (C8), 36.31 (C2‴), 37.73 (C5), 38.16 (C3‴), 39.54 (C1), 60.1 (C4), 62.40 (C2), 64.38 (N**CH**_**2**_Ph), 71.20 (C1′), 72.63 (C9), 127.92 (C4 Ph), 128.87 (C1″), 129.26, 129.75 (C2 Ph, C3 Ph, C5 Ph, C6 Ph), 130.48 (C5″), 131.12 (C3″), 132.00 (C6″), 133.97 (C2″), 134.48 (C4″), 140.5 (C1 Ph), 165.7 (ester), 174.9 (C4‴), 181.6 (C1‴).

#### ((9*R*)-9-Hydroxy-3-(2-phenethyl)-3-azabicyclo[3.3.1]nonan-1-yl)methyl 2-((*S*)-3-Methyl-2,5-dioxopyrrolidin-1-yl)benzoate (**17**)

Compound **6** (0.634 mmol, 200 mg) was reduced using LAH (1.585 mmol, 60.2 mg) as described for compound **9**. The crude product was used for the esterification step without purification. Compound **13** (0.2 mmol, 46.6 mg) was stirred with DCC (0.2 mmol, 41.6 mg, 99%) and DMAP (0.02 mmol, 2.5 mg, 99%) in anhydrous acetonitrile under nitrogen gas at 40 °C for 20 min, and then the crude amino alcohol (55 mg) was added. The reaction monitored by TLC and stopped after 24 h. The mixture was concentrated under vacuum and purified over column chromatography with 5% MeOH in DCM to yield the title compound **17** (25 mg, 26%) as a yellow oil. R_f_ = 0.54 (5% MeOH in DCM). HRMS (ESI): *m*/*z* calcd. for C_29_H_35_N_2_O_5_: 491.2546, found: 491.2549 [M + H]^+^. ^1^H NMR (500 MHz, CD_3_OD): δ (ppm) = 1.37–1.52 (m, 6H, H5‴, H6_eq_, H7_eq_, H8_eq_), 1.67–1.80 (m, 1H, H8_ax_), 1.91 (br s, 1H, H5), 1.97–2.08 (m, 1H, H6_ax_), 2.09–2.18 (m, 1H, H7_ax_), 2.34–2.42 (m, 1H, H2_ax_), 2.45–2.60 (m, 2H, H4_ax_ and H3‴A), 2.63–2.73 (m, 2H, N**CH**_**2**_CH_2_Ph), 2.80–2.89 (m, 2H, NCH_2_**CH**_**2**_Ph), 3.02–3.16 (m, 2H, H2‴ and H3‴B), 3.18–3.28 (m, 2H, H2_eq_ and H4_eq_), 3.63–3.72 (m, 1H, H9), 4.00–4.12 (m, 2H, H1′ A and B), 7.12–7.30 (m, 5H, NCH_2_CH_2_**Ph**), 7.35 (d, *J* = 7.5 Hz, 1H, H3″), 7.61 (td, *J* = 7.5, 1.3 Hz, 1H, H5″), 7.74 (td, *J* = 7.5, 1.5 Hz, 1H, H4″), 8.13 (dd, *J* = 7.5, 1.6 Hz, 1H, H6″). ^13^C NMR (125 MHz, CDCl_3_): δ (ppm) = 16.1 (5‴), 20.6 (C7), 24.3 (C6), 27.2 (C8), 33.45 (NCH_2_**CH**_**2**_Ph), 35.76 (C2‴), 36.62 (C5), 37.48 (C3‴), 39.58 (C1), 59.46 (C4), 60.81 (N**CH**_**2**_CH_2_Ph), 61.30 (C2), 70.00 (C1′), 70.85 (C9), 127.07 (C4 Ph), 128.96 (C1″), 129.29, 129.73 (C2 Ph, C3 Ph, C5 Ph, C6 Ph), 130.50 (C5″), 131.04 (C3″), 132.15 (C6″), 133.26 (C2″), 134.47 (C4″), 139.67 (C1 Ph), 164.5 (ester), 177.9 (C4‴), 180.8 (C1‴).

#### ((9*R*)-9-Hydroxy-3-(3-phenylpropyl)-3-azabicyclo[3.3.1]nonan-1-yl)methyl 2-((*S*)-3-Methyl-2,5-dioxopyrrolidin-1-yl)benzoate (**18**)

Compound **7** (0.91 mmol, 300 mg) was reduced using LAH (2.28 mmol, 86.3 mg) as described for compound **9**. The crude product was used for the esterification step without purification. Compound **13** (0.17 mmol, 40.3 mg) was stirred with DCC (0.17 mmol, 36 mg, 99%) and DMAP (0.017 mmol, 2.1 mg, 99%) in anhydrous acetonitrile under nitrogen gas at 40 °C for 20 min, and then the crude amino alcohol (50 mg) was added. The reaction was monitored by TLC and stopped after 24 h. The mixture was concentrated under vacuum and purified over column chromatography with 5% MeOH in DCM to yield the title compound **18** (26 mg, 30%) as a yellow oil. R_f_ = 0.57 (5% MeOH in DCM). HRMS (ESI): *m*/*z* calcd. for C_30_H_37_N_2_O_5_: 505.2703, found: 505.2701 [M + H]^+^. ^1^H NMR (500 MHz, CD_3_OD): δ (ppm) = 1.41 (d, *J* = 7.5 Hz, H5‴), 1.49–1.74 (m, 3H, H6_eq_, H7_eq_, H8_eq_), 1.66–1.88 (m, 1H, H8_ax_), 1.89–1.96 (m, 1H, H5), 1.96–2.02 (m, 2H, NCH_2_**CH**_**2**_CH_2_Ph), 2.04–2.12 (m, 1H, H6_ax_), 2.13–2.26 (m, 1H, H7_ax_), 2.29–2.39 (m, 1H, H2_ax_), 2.42–2.55 (m, 2H, H4_ax_ and H3‴A), 2.57–2.63 (m, 2H, N**CH**_**2**_CH_2_CH_2_Ph), 2.64–2.74 (m, 2H, NCH_2_CH_2_**CH**_**2**_Ph), 3.01–3.06 (m, 1H, H2_eq_), 3.05–3.16 (m, 3H, H4_eq_, H2‴ and H3‴B), 3.63–3.68 (m, 1H, H9), 4.04–4.16 (m, 2H, H1′ A and B), 7.12–7.30 (m, 5H, NCH_2_CH_2_CH_2_**Ph**), 7.34 (dd, *J* = 7.7, 1.3 Hz, 1H, H3″), 7.58 (td, *J* = 7.7, 1.3 Hz, 1H, H5″), 7.72 (td, *J* = 7.7, 1.6 Hz, 1H, H4″), 8.10 (dd, *J* = 7.7, 1.6 Hz, 1H, H6″). ^13^C NMR (125 MHz, CDCl_3_): δ (ppm) = 16.5 (5‴), 21.0 (C7), 24.3 (C6), 27.6 (C8), 32.0 (NCH_2_**CH**_**2**_CH_2_Ph), 34.0 (NCH_2_CH_2_**CH**_**2**_Ph), 36.64 (C2‴), 36.72 (C5), 38.10 (C3‴), 39.61 (C1), 59.88 (C4), 60.53 (N**CH**_**2**_CH_2_CH_2_Ph), 61.42 (C2), 70.57 (C1′), 71.67 (C9), 126.98 (C4 Ph), 128.87 (C1″), 129.33, 129.56 (C2 Ph, C3 Ph, C5 Ph, C6 Ph), 130.43 (C5″), 131.06 (C3″), 132.36 (C6″), 133.57 (C2″), 134.43 (C4″), 143.0 (C1 Ph), 166.4 (ester), 177.4 (C4‴), 182.0 (C1‴).

#### ((9*R*)-9-Hydroxy-3-(4-phenylbutyl)-3-azabicyclo[3.3.1]nonan-1-yl)methyl 2-((*S*)-3-Methyl-2,5-dioxopyrrolidin-1-yl)benzoate (**19**)

Compound **8** (0.58 mmol, 200 mg) was reduced using LAH (1.54 mmol, 55 mg) as described for compound **9**. The crude product was used for the esterification step without purification. Compound **13** (0.158 mmol, 36.8 mg) was stirred with DCC (0.158 mmol, 32.9 mg, 99%) and DMAP (0.0158 mmol, 1.9 mg, 99%) in anhydrous acetonitrile under nitrogen gas at 40 °C for 20 min, and then the crude amino alcohol (48 mg) was added. The reaction was monitored by TLC and stopped after 24 h. The mixture was concentrated under vacuum and purified over column chromatography with 5% MeOH in DCM to yield the title compound **19** (20 mg, 25%) as a yellow oil. R_f_ = 0.61 (5% MeOH in DCM). HRMS (ESI): *m*/*z* calcd. for C_31_H_39_N_2_O_5_: 519.2859, found: 519.2851 [M + H]^+^. ^1^H NMR (500 MHz, CD_3_OD): δ (ppm) = 1.41 (d, *J* = 7.1 Hz, H5‴), 1.46–1.74 (m, 3H, H6_eq_, H7_eq_, H8_eq_), 1.79–1.97 (m, 4H, H5, H8_ax_ and NCH_2_**CH**_**2**_CH_2_CH_2_Ph), 1.98–2.10 (m, 3H, H6_ax_ and NCH_2_CH_2_**CH**_**2**_CH_2_Ph), 2.17–2.29 (m, 1H, H7_ax_), 2.33–2.42 (m, 1H, H2_ax_), 2.43–2.52 (m, 2H, H4_ax_ and H3‴A), 2.54–2.61 (m, 2H, N**CH**_**2**_CH_2_CH_2_CH_2_Ph), 2.62–2.77 (m, 2H, NCH_2_CH_2_CH_2_**CH**_**2**_Ph), 2.99–3.05 (m, 1H, H2_eq_), 3.06–3.16 (m, 3H, H4_eq_, H2‴ and H3‴B), 3.62–3.66 (m, 1H, H9), 4.01–4.18 (m, 2H, H1′ A and B), 7.10–7.30 (m, 5H, NCH_2_CH_2_CH_2_**Ph**), 7.34 (dd, *J* = 7.9, 1.3 Hz, 1H, H3″), 7.58 (td, *J* = 7.9, 1.3 Hz, 1H, H5″), 7.72 (td, *J* = 7.9, 1.6 Hz, 1H, H4″), 8.10 (dd, *J* = 7.9, 1.6 Hz, 1H, H6″). ^13^C NMR (125 MHz, CDCl_3_): δ (ppm) = 16.5 (5‴), 21.1 (C7), 24.4 (C6), 28.8 (C8), 32.0 (NCH_2_CH_2_**CH**_**2**_CH_2_Ph), 34.71 (NCH_2_**CH**_**2**_CH_2_CH_2_Ph), 36.33 (C2‴), 36.59 (NCH_2_CH_2_CH_2_**CH**_**2**_Ph), 37.19 (C5), 38.05 (C3‴), 39.54 (C1), 60.00 (C4), 60.48 (N**CH**_**2**_CH_2_CH_2_CH_2_Ph), 61.26 (C2), 70.35 (C1′), 71.70 (C9), 127.07 (C4 Ph), 128.50 (C1″), 129.35, 129.50 (C2 Ph, C3 Ph, C5 Ph, C6 Ph), 130.45 (C5″), 131.05 (C3″), 132.35 (C6″), 133.35 (C2″), 134.47 (C4″), 143.0 (C1 Ph), 166.5 (ester), 177.4 (C4‴), 181.9 (C1‴).

#### ((9*R*)-9-Hydroxy-3-methyl-3-azabicyclo[3.3.1]nonan-1-yl)methyl 2-Acetamidobenzoate (**20**)

Compound **3** (0.89 mmol, 200 mg) was reduced using LAH (1.78 mmol, 84.2 mg) as described for compound **9**. The crude product was used for the esterification step without purification. Compound **11** (0.189 mmol, 33.8 mg) was stirred with DCC (0.189 mmol, 39.4 mg, 99%) and DMAP (0.0189 mmol, 2.3 mg, 99%) in anhydrous acetonitrile under nitrogen gas at 40 °C for 20 min, and then the crude amino alcohol (35 mg) was added. The reaction was monitored by TLC and stopped after 24 h. The mixture was concentrated under vacuum and purified over column chromatography with 5% MeOH in DCM to yield the title compound **20** (29 mg, 45%) as a yellow oil. R_f_ = 0.6 (5% MeOH in DCM). HRMS (ESI): *m*/*z* calcd. for C_19_H_27_N_2_O_4_: 347.1971, found: 347.1969 [M + H]^+^. ^1^H NMR (500 MHz, CD_3_OD): δ (ppm) = 1.43–1.59 (m, 3H, H6_eq_, H7_eq_, H8_eq_), 1.78–1.83 (m, 1H, H8_ax_), 1.86 (br s, 1H, H5), 1.99–2.08 (m, 1H, H6_ax_), 2.18 (s, 3H, N–CH_3_), 2.19–2.22 (m, 4H, NHCO**CH**_**3**_, H2_ax_), 2.32 (d, *J* = 11.4 Hz,1H, H4_ax_), 2.53–2.61 (m, 1H, H7_ax_), 2.92 (d, *J* = 11.3 Hz, 1H, H2_eq_), 2.95 (d, *J* = 11.4 Hz, 1H, H4_eq_), 3.67 (d, *J* = 3.8 Hz, 1H, H9), 4.08 (d, *J* = 11.0 Hz, 1H, H1′A), 4.14 (d, *J* = 11.0 Hz, 1H, H1′B), 7.19 (td, *J* = 7.7, 1.3 Hz, 1H, H5″), 7.58 (ddd, *J* = 8.6, 7.7, 1.3 Hz, 1H, H4″), 8.05 (dd, *J* = 7.7, 1.3 Hz, 1H, H6″), 8.46 (d, *J* = 8.6 Hz, 1H, H3″). ^13^C NMR (125 MHz, CDCl_3_): δ (ppm) = 21.3 (C7), 24.48 (NHCO**CH**_**3**_), 24.77 (C6), 28.0 (C8), 37.35 (C5), 39.44 (C1), 46.1 (NCH_3_), 61.9 (C4), 64.2 (C2), 71.09 (C1′), 72.00 (C9), 116.6 (C1″), 121.5 (C3″), 124.1 (C5″), 131.3 (C6″), 134.8 (C4″), 140.4 (C2″), 167.5 (ester), 169.9 (amide).

#### ((9*R*)-3-Ethyl-9-hydroxy-3-azabicyclo[3.3.1]nonan-1-yl)methyl 2-Acetamidobenzoate (**21**)

Compound **11** (0.8 mmol, 143.9 mg) was stirred with DCC (0.8 mmol, 167 mg, 99%) and DMAP (0.08 mmol, 9.9 mg, 99%) in anhydrous acetonitrile under nitrogen gas at 40 °C for 20 min, and then compound **9** (160 mg) was added. The reaction was monitored by TLC and stopped after 24 h. The mixture was concentrated under vacuum and purified over column chromatography with 5% MeOH in DCM to yield the title compound **21** (130 mg, 45%) as a yellow oil. R_f_ = 0.64 (5% MeOH in DCM). HRMS (ESI): *m*/*z* calcd. for C_20_H_29_N_2_O_4_: 361.2127, found: 361.2122 [M + H]^+^. ^1^H NMR (500 MHz, CD_3_OD): δ (ppm) = 1.20 (t, *J* = 7.2 Hz, 3H, NCH_2_**CH**_**3**_), 1.52–1.66 (m, 3H, H6_eq_, H7_eq_, H8_eq_), 1.81–1.92 (m, 1H, H8_ax_), 1.99–2.16 (m, 2H, H5 and H6_ax_), 2.20 (s, 3H, NHCO**CH**_**3**_), 2.24–2.34 (m, 1H, H7_ax_), 2.58–2.80 (m, 4H, H2_ax_, H4_ax_ and N**CH**_**2**_CH_3_), 3.27–3.36 (m, 2H, H2_eq_ and H4_eq_), 3.82 (d, *J* = 3.5 Hz, 1H, H9), 4.09 (d, *J* = 11.2 Hz, 1H, H1′A), 4.21 (d, *J* = 11.2 Hz, 1H, H1′B), 7.20 (t, *J* = 7.7 Hz, 1H, H5″), 7.58 (ddd, *J* = 8.7, 7.7, 1.7 Hz, 1H, H4″), 8.07 (dd, *J* = 7.7, 1.3 Hz, 1H, H6″), 8.42 (d, *J* = 8.7 Hz, 1H, H3″). ^13^C NMR (125 MHz, CDCl_3_): δ (ppm) = 11.2 (NCH_2_**CH**_**3**_), 20.5 (C7), 23.76 (C6), 24.53 (NHCO**CH**_**3**_), 26.9 (C8), 36.4 (C5), 39.4 (C1), 54.3 (N**CH**_**2**_CH_3_), 58.90 (C4), 60.63 (C2), 69.88 (C9), 70.16 (C1′), 118.2 (C1″), 121.8 (C3″), 123.9 (C5″), 131.6 (C6″), 135.0 (C4″), 141.5 (C2″), 169.0 (ester), 171.5 (amide).

### Electrophysiological Methods

Oocyte expression studies employed the human α7 nAChR subunit in plasmid pSP64GL.^32^ Oocytes were isolated from adult female *Xenopus laevis* and defolliculated by treatment with collagenase (2.5 mg/mL; Gibco, ThermoFisher Scientific) in calcium-free Barth’s solution containing 88 mM NaCl, 2.4 mM NaHCO_3_, 1 mM KCl, 0.82 mM MgSO_4_, and 15 mM Tris, pH 7.5, as described previously.^33^ Heterologous expression was achieved by cytoplasmic injection of *in vitro* transcribed cRNA. Prior to *in vitro* synthesis of cRNA plasmid, cDNA was linearized by restriction enzyme digestion and purified with QIAQuik PCR purification kit (Qiagen). *In vitro* synthesis of cRNA was performed using mMessage mMachine SP6 transcription kit (ThermoFisher Scientific).

Oocytes were injected with approximately 9 ng of cRNA using a Drummond variable volume microinjector. After injection, oocytes were incubated at 14 °C in calcium-containing Barth’s solution (composition as above but with 0.77 mM CaCl_2_) supplemented with antibiotics (100 units/mL penicillin, 100 μg/mL streptomycin, 4 μg/mL kanamycin, and 50 μg/mL tetracycline). Experiments were performed on oocytes after 3 to 5 d of incubation. Oocytes were placed in a recording chamber and continuously perfused with a modified Ringer’s solution (115 mM NaCl, 2.5 mM KCl, 1.8 mM BaCl_2_, and 10 mM HEPES, pH 7.3) with a flow rate of approximately 15 mL/min. Two-electrode voltage-clamp recordings were performed using a Warner Instruments OC-725C amplifier (Harvard Apparatus) with the oocyte membrane potential held at −60 mV, as described previously.^34,35^ Application of compounds was controlled by LabChart software (AD Instruments) using a BPS-8 solenoid valve solution exchange system (ALA Scientific Inc.). Compounds were preapplied for 2 min before coapplication with EC_50_ concentration of agonist (100 μM ACh) and normalized to responses to the EC_50_ concentration of agonist in the absence of the compound on the same oocyte. Data are presented as mean ± SEM of at least three independent experiments, that were conducted on separate oocytes. For multiple comparisons, statistical significance was determined with an unpaired one-way analysis of variance (ANOVA).

## Supporting Information Available

^1^H and ^13^C NMR spectra of a mixture of epimers from reduction of cyclohexanone **4**, ^13^C NMR spectra of chiral **13**, NOESY of analogues **14** and **21**, expanded ^1^H NMR spectra at 1′ on **14**, **15**, **20**, and **21**, and HPLC traces for target compounds **14**–**21** (PDF)
