# Supplementary material for: Synthesis and Antagonist Activity of Methyllycaconitine Analogues on Human α7 Nicotinic Acetylcholine Receptors
Source: ACS Bio Med Chem Au. 2023 Feb 14;3(2):147–57. doi: 10.1021/acsbiomedchemau.2c00057 (PMC10119942; doi:10.1021/acsbiomedchemau.2c00057)
Supplement: Supplementary file 1 — bg2c00057_si_001.pdf [file bg2c00057_si_001.pdf]

Supplementary Information

Synthesis and Antagonist Activity of Methyllycaconitine Analogues on Human α7 Nicotinic Acetylcholine Receptors

Ashraf M. A. Qasem, Michael G. Rowan, Victoria R. Sanders,[a] Neil S. Millar,[a] and Ian S. Blagbrough*

School of Pharmacy, University of Bath, Bath BA2 7AY, U.K.

[a] Department of Neuroscience, Physiology and Pharmacology, University College London, Gower Street, London WC1E 6BT, U.K.

*Corresponding Author: tel.: 1225-386795; Email: prsisb@bath.ac.uk

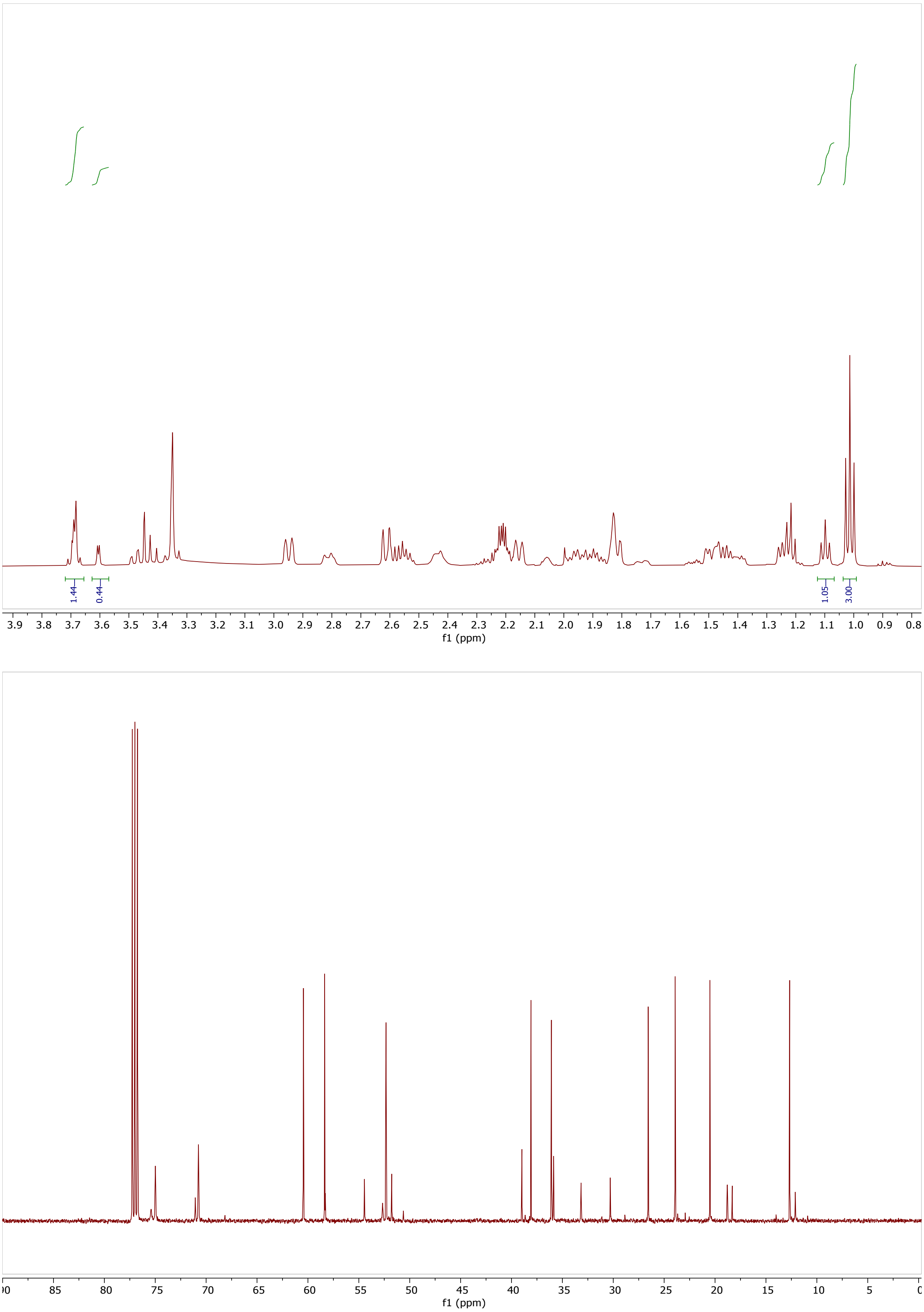

Figure S1. The ^{1}H NMR (500 MHz) (upper) and ^{13}C NMR (125 MHz) (lower) in $CDCl_3$ of the epimeric mixture after reduction of the cyclohexanone in AE-bicycle **4**.

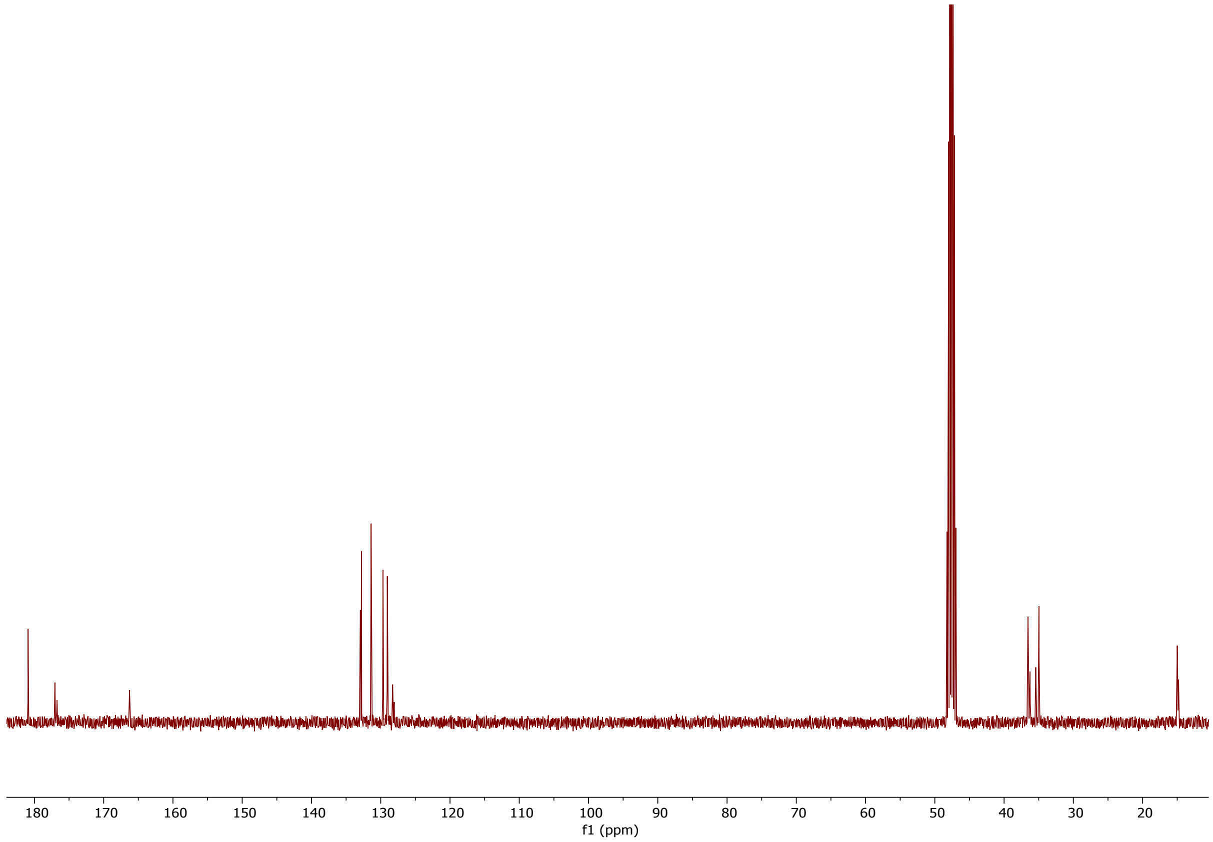

Figure S2. ^{13}C NMR (125 MHz) in CD_3OD of **13** at 25 ^{o}C (298 K).

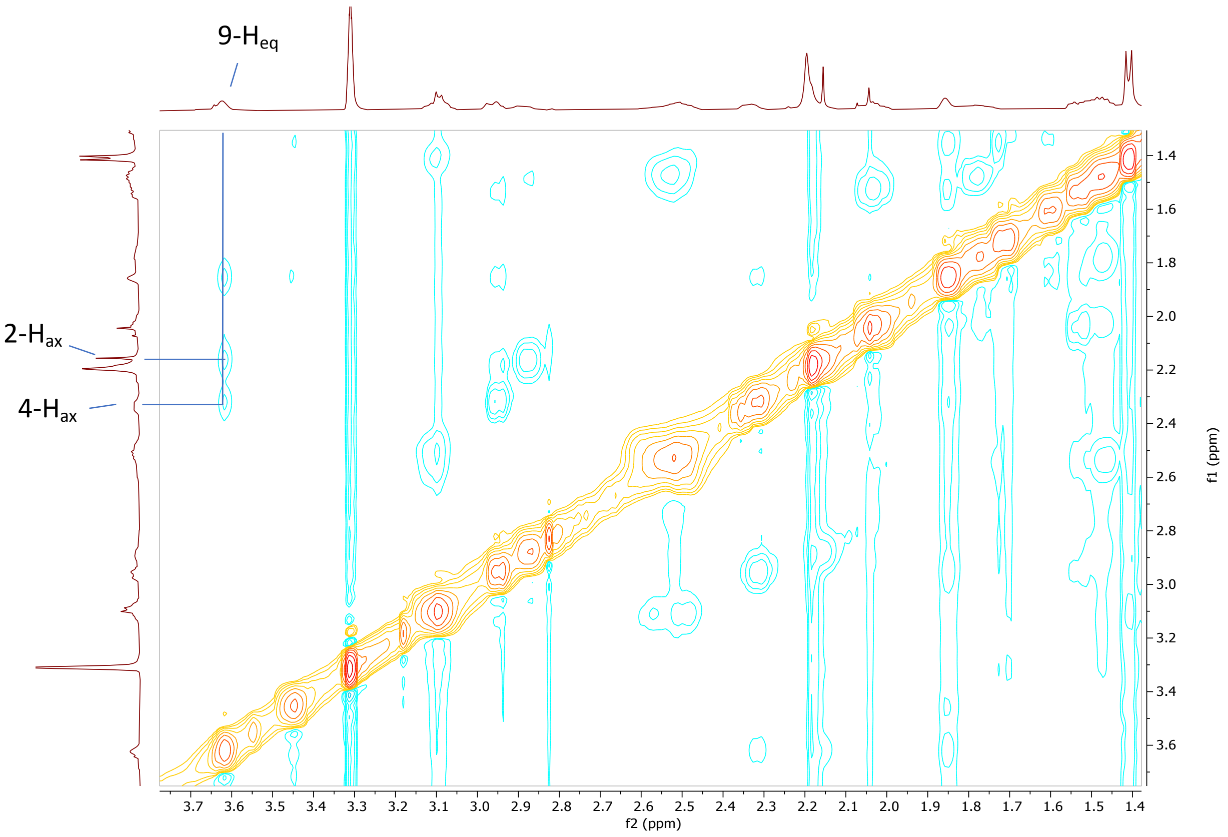

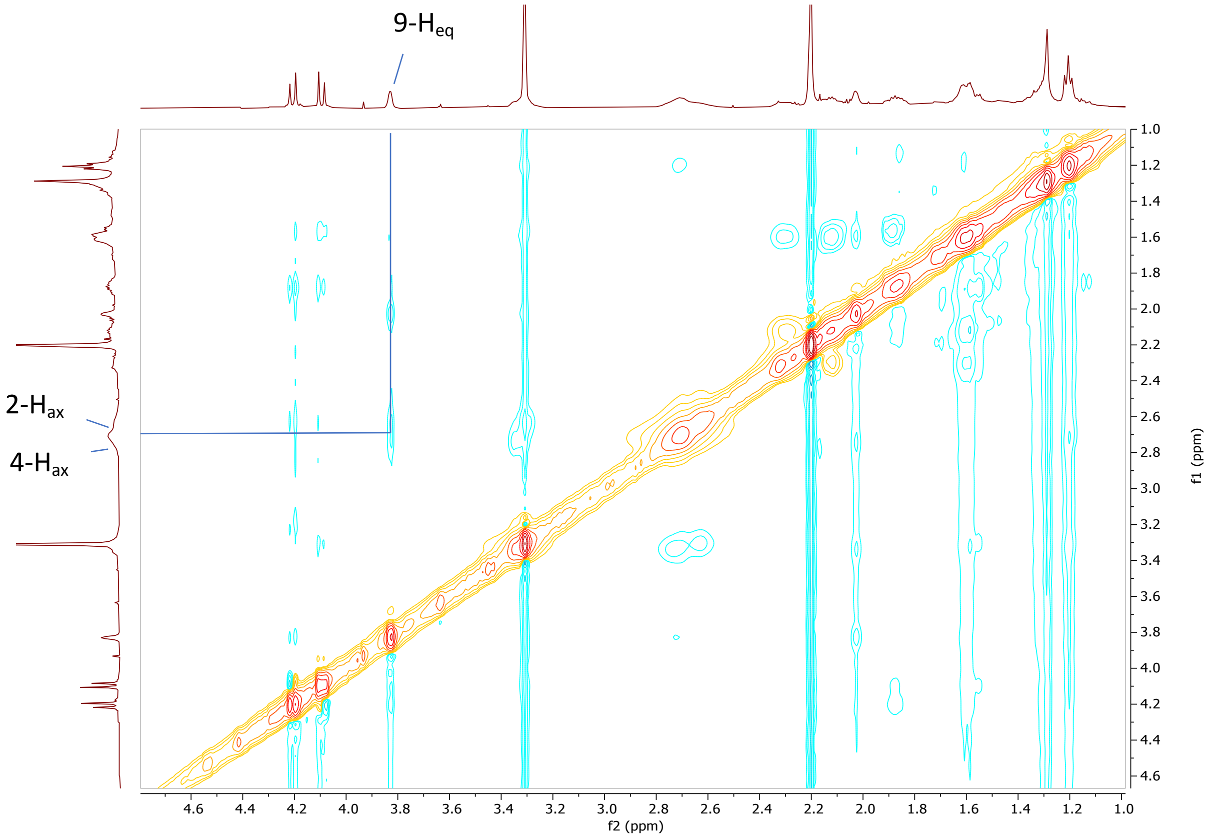

Figure S3. NOESY correlation between 9-H$_{eq}$ and 2-H$_{ax}$, 4-H$_{ax}$ in analogues **14** (upper) and **21** (lower).

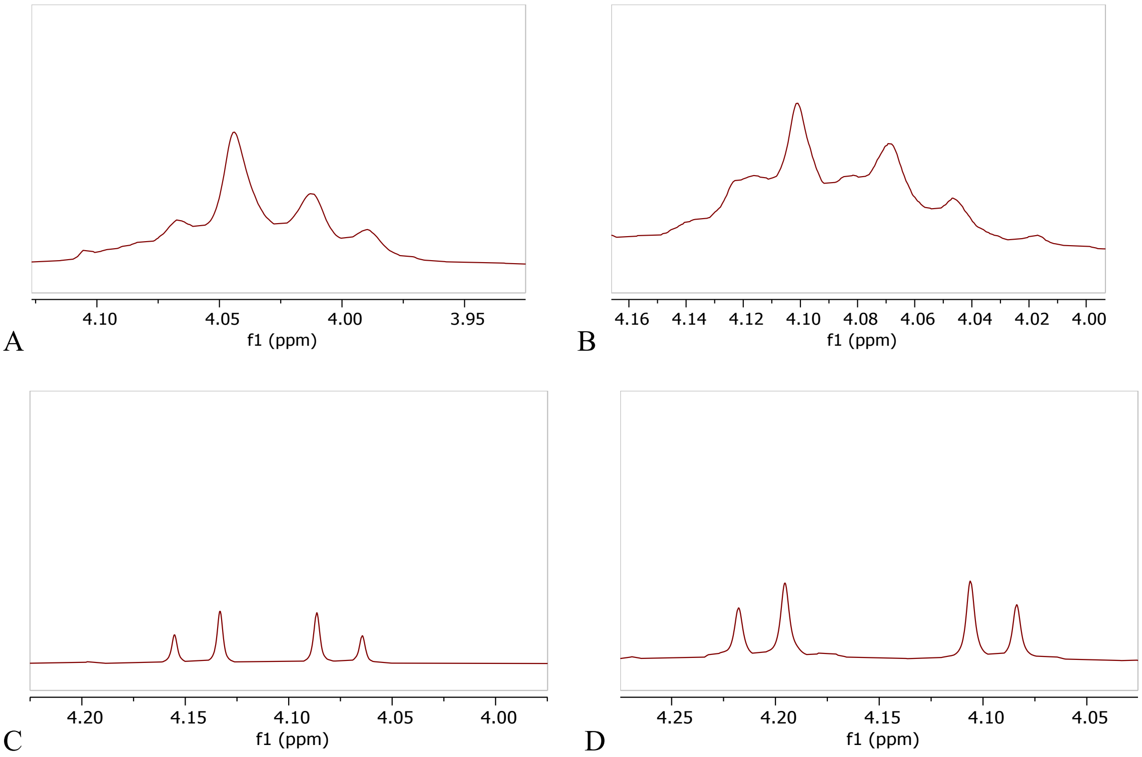

Figure S4. ^{1}H NMR signal at position 1′ in analogues **14** and **15** (A and B respectively) and **20** and **21** (C and D respectively).

HPLC purity assays of analogues 14-21

((9*R*)-9-Hydroxy-3-methyl-3-azabicyclo[3.3.1]nonan-1-yl)methyl 2-((*S*)-3-methyl-2,5-dioxopyrrolidin-1-yl)benzoate (**14**)

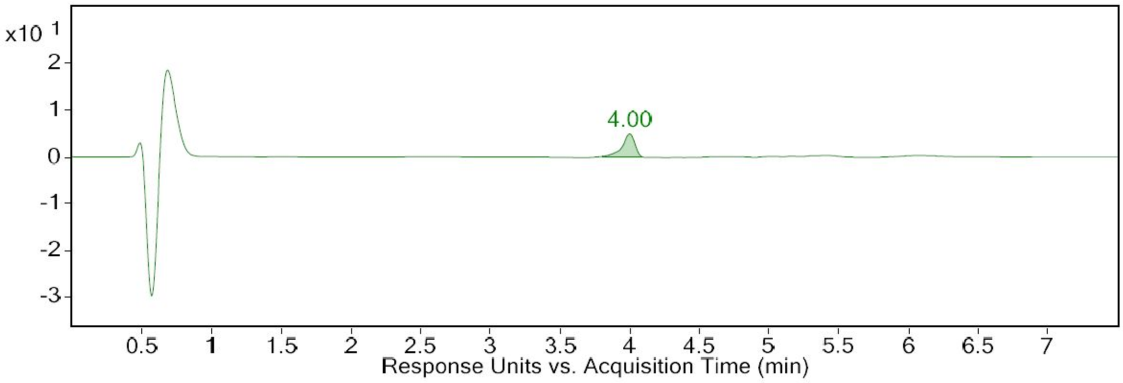

Integration Peak List

Peak	Start	RT	End	Height	Area	Area %
1	3.8	4	4.09	4.9	30.48	100

((9*R*)-3-Ethyl-9-hydroxy-3-azabicyclo[3.3.1]nonan-1-yl)methyl 2-((*S*)-3-methyl-2,5-dioxopyrrolidin-1-yl)benzoate (**15**)

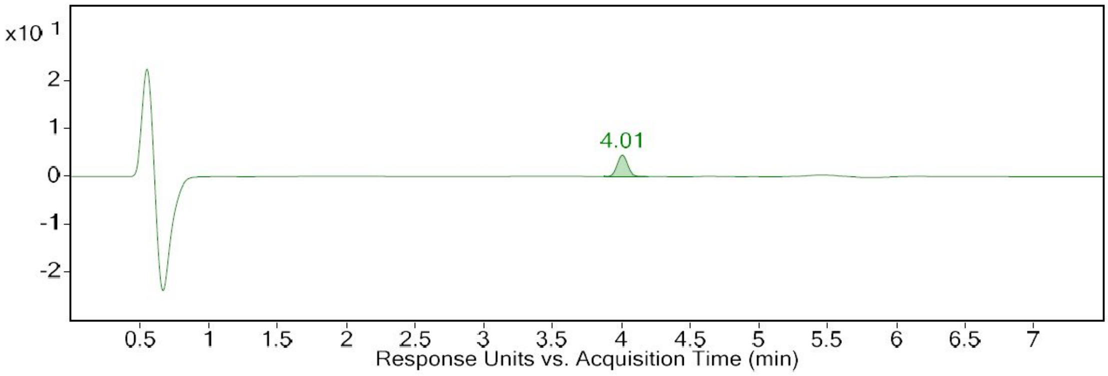

Integration Peak List

Peak	Start	RT	End	Height	Area	Area %
1	3.88	4.01	4.19	4.45	23.58	100

((9*R*)-3-Benzyl-9-hydroxy-3-azabicyclo[3.3.1]nonan-1-yl)methyl 2-((*S*)-3-methyl-2,5-dioxopyrrolidin-1-yl)benzoate (**16**)

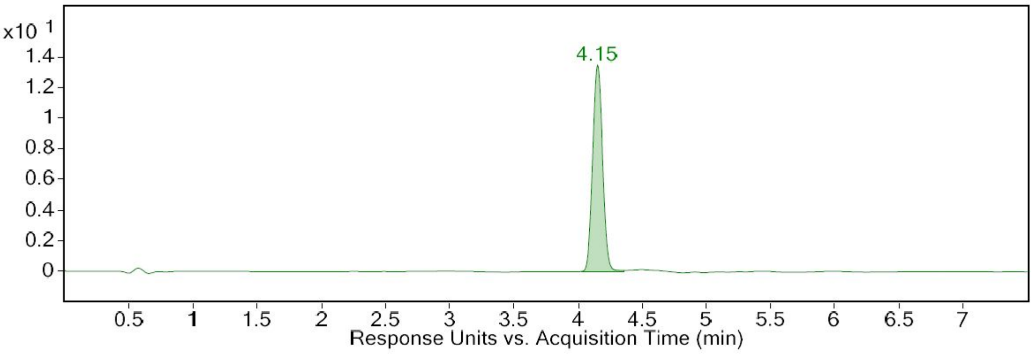

Integration Peak List

Peak	Start	RT	End	Height	Area	Area %
1	4.02	4.15	4.36	13.53	72.99	100

((9*R*)-9-Hydroxy-3-(2-phenethyl)-3-azabicyclo[3.3.1]nonan-1-yl)methyl 2-((*S*)-3-methyl-2,5-dioxopyrrolidin-1-yl)benzoate (**17**)

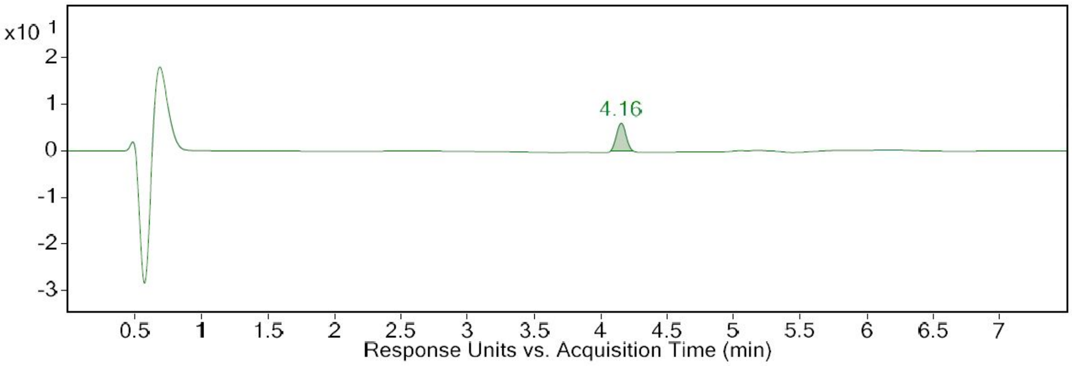

Integration Peak List

Peak	Start	RT	End	Height	Area	Area %
1	4.07	4.16	4.25	6.03	29.88	100

((9*R*)-9-Hydroxy-3-(3-phenylpropyl)-3-azabicyclo[3.3.1]nonan-1-yl)methyl 2-((*S*)-3-methyl-2,5-dioxopyrrolidin-1-yl)benzoate (**18**)

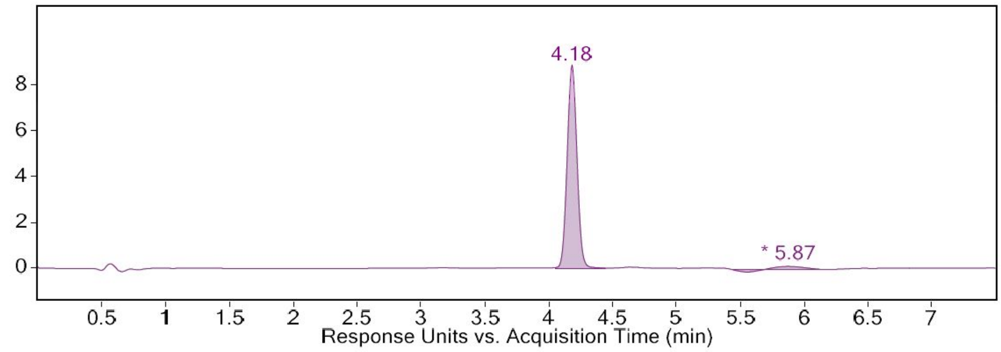

Integration Peak List

Peak	Start	RT	End	Height	Area	Area %
1	4.05	4.18	4.44	8.92	47.68	100
2	5.43	5.87	6.17	0.14	1.17	2.46

((9*R*)-9-Hydroxy-3-(4-phenylbutyl)-3-azabicyclo[3.3.1]nonan-1-yl)methyl 2-((*S*)-3-methyl-2,5-dioxopyrrolidin-1-yl)benzoate (**19**)

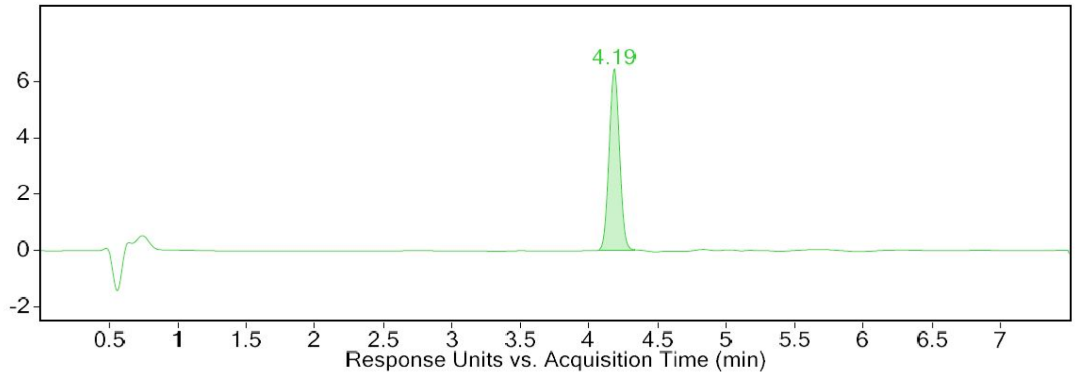

Integration Peak List

Peak	Start	RT	End	Height	Area	Area %
1	4.07	4.19	4.33	6.45	34.2	100

((9*R*)-9-Hydroxy-3-methyl-3-azabicyclo[3.3.1]nonan-1-yl)methyl 2-acetamidobenzoate (**20**)

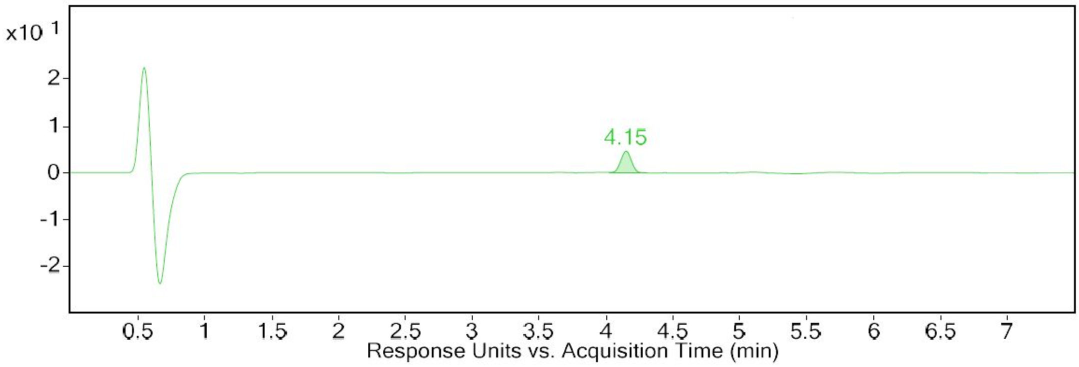

Integration Peak List

Peak	Start	RT	End	Height	Area	Area %
1	4.02	4.15	4.3	4.63	25.58	100

((9*R*)-3-Ethyl-9-hydroxy-3-azabicyclo[3.3.1]nonan-1-yl)methyl 2-acetamidobenzoate (**21**)

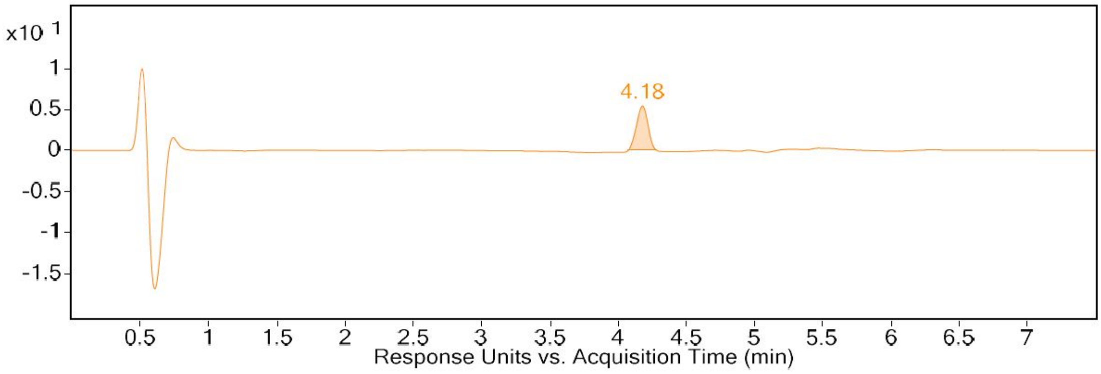

Integration Peak List

Peak	Start	RT	End	Height	Area	Area %
1	4.07	4.18	4.28	5.4	31.07	100
